# Optimizing electric vehicle powertrains peak performance with robust predictive direct torque control of induction motors: a practical approach and experimental validation

**DOI:** 10.1038/s41598-024-65988-0

**Published:** 2024-06-28

**Authors:** Amel Kasri, Kamel Ouari, Youcef Belkhier, Mohit Bajaj, Ievgen Zaitsev

**Affiliations:** 1 Université de Bejaia, Faculté de Technologie, Laboratoire de Technologie Industrielle et de l’Information, 06000 Bejaia, Algeria; 2 Institut de Recherche de l’Ecole Navale (EA 3634, IRENav), French Naval Academy, 29240 Brest, France; 3 Department of Electrical Engineering, Graphic Era (Deemed to be University), Dehradun, 248002 India; 4 Hourani Center for Applied Scientific Research, Al-Ahliyya Amman University, Amman, Jordan; 5 Graphic Era Hill University, Dehradun, 248002 India; 6 Department of Theoretical Electrical Engineering and Diagnostics of Electrical Equipment, Institute of Electrodynamics, National Academy of Sciences of Ukraine, Peremogy, 56, Kyiv-57, 03680 Ukraine; 7 Center for Information-Analytical and Technical Support of Nuclear Power Facilities Monitoring of the National Academy of Sciences of Ukraine, 34-A, Akademika Palladina Avenue, Kyiv, Ukraine

**Keywords:** Robust model predictive control, Direct torque control, Electric vehicle powertrains, Induction motor drives, Robust speed controller, Energy science and technology, Engineering, Mathematics and computing

## Abstract

Enhancing the efficiency of the electric vehicle’s powertrain becomes a crucial focus, wherein the control system for the traction motor plays a significant role. This paper presents a novel electric vehicle traction motor control system based on a robust predictive direct torque control approach, an improved version of the conventional DTC, where the traditional switching table and the hysteresis regulators are substituted with a predictive block based on an optimization algorithm. Additionally, a robust predictive speed loop regulator is employed instead of the proportional-integral regulator, which integrates a new cost function with a finite horizon, incorporating integral action into the control law based on a Taylor series expansion. This technique’s primary benefit is its independence from the necessity to measure and observe external disturbances, as well as uncertainties related to parameters. The effectiveness of the suggested system was confirmed through simulation and experimental results under the OPAL-RT platform. The findings indicate that the proposed approach outperforms the conventional method in terms of rejecting disturbances, exhibiting robustness to variations in parameters, and minimizing torque ripple.

## Introduction

Most cities and industries worldwide rely on transportation and vehicular movement, where the predominant type of vehicle uses the internal combustion engine, which, unfortunately, produces tailpipe emissions. These emissions contribute to urban air pollution and play a role in generating greenhouse gases^1^. Furthermore, an escalating usage of fossil fuels raises undeniable concerns about the eventual exhaustion of finite resources. Efforts need to be implemented to alleviate the environmental impacts of the transportation sector, addressing concerns related to the ecosystem, as well as the exhaustion of worldwide natural resources^2^. Addressing the current environmental challenges fundamentally involves the exploration and advancement of electric vehicles (EVs), which rely on batteries for power. The substantial advantages associated with this type of vehicle encompass zero emissions of gases or pollutants, high efficiency, freedom from dependence on petroleum, and a quiet, smooth operation that contributes to low noise pollution^1^.

The incorporation of various mechanical design principles enables the enhancement of electric vehicle performance. Nevertheless, the optimization of EV powertrains offers the potential for added effectiveness and extended driving range, specifically emphasizing the electrification of the EV powertrain. A key facet of such optimization involves improving the electric vehicle’s driving range by employing highly efficient electric motors^3^. Three-phase induction machines stand out among various electric motor types and have increasingly replaced DC machines due to their impressive performance, reliability, straightforward construction, cost-effectiveness, and easy maintenance^4^. However, these numerous benefits come with challenges as the nature of the machine’s dynamic behavior is frequently intricate because its modeling yields a set of nonlinear, tightly coupled, and multivariable equations. Moreover, certain state variables, like flux, are not directly measurable^5^. These limitations call for the implementation of advanced control algorithms to effectively manage the real-time control of torque and flux in these machines.

For numerous years, research endeavors have been focused on tackling the control challenges associated with induction machines (IMs) and devising resilient and efficient control strategies. In this context, various approaches have been created. One of these approaches is the field-oriented control (FOC) technique, designed to manage fluctuating torque. Employing this technique aligns the performance of an IM closely with that of a DC machine, introducing a decoupling or separation of flux and torque^4^. While this decoupling results in a rapid torque response and enables a broad range of speed control, it comes with notable drawbacks, and one of its core limitations is associated with coordinate transformation. The flux angle is required for the conversion to occur, a challenging parameter to measure directly due to the difficulty of placing a flux sensor inside the machine’s air gap. Additionally, another limitation is associated with the machine’s susceptibility to variations in parameters^6^. On the other hand, direct torque control (DTC) represents an alternative method that guarantees independent and isolated control of flux and torque, in contrast to FOC, this control operations exclusively within a stationary frame^7^. Furthermore, the DTC produces the gating signals for the inverter by directly utilizing a lookup switching table, eliminating the need for modulation such as PWM. Despite having fewer model parameters compared to FOC, DTC yields superior torque and flux responses. In essence, DTC is characterized by its simplicity and remarkably rapid response, making it well-suited for high-performance drive applications^8^. Nonetheless, the utilization of hysteresis controllers introduces notable fluctuations in both flux and electromagnetic torque, leading to the generation of mechanical vibrations and unwanted acoustic noise. Consequently, this results in a decline in machine performance. The fluctuating switching frequency additionally adds to losses in switching and distortions in current, potentially reducing the output power’s quality^9^. Additionally, DTC faces substantial challenges when integrated with a PI regulator. The motor needs to exhibit a rapid and precise dynamic response during operation, without any static error. Moreover, frequent and substantial changes in load torque, coupled with common model uncertainties in parameters, can contribute to system instability^10^.

Recently, significant strides have been achieved in improving the efficacy of direct torque control. Integrating advanced mathematical methods has resulted in a fundamental shift in resilient and nonlinear controlling methodologies, effectively overcoming the inherent limitations associated with traditional linear control methods for electric motors. The Backstepping technique proposed in^11^ is an approach designed to stabilize the system by systematically synthesizing controllers using Lyapunov’s approach. Despite its precision, this approach exhibits limited resistance to parameter uncertainties. Another robust and nonlinear strategy, sliding mode control (SMC), has been suggested to enhance classical direct torque control. SMC is known for its excellent dynamic behavior and considerable insensitivity to parameter and disturbance fluctuations^12^. Unfortunately, a drawback of this approach is the occurrence of the “chattering” phenomenon, leading to elevated harmonics, thereby restricting its applicability in a broad variety of viable applications. Numerous studies, such as^13^, have suggested integrating DTC with artificial neural networks (ANN). In these proposals, the ANN technique substitutes the traditional hysteresis regulators and DTC’s switching table. Nevertheless, implementing this combination necessitates processors with parallel processing capabilities based on their architecture. Additionally, determining an optimal network structure involves multiple trial-and-error iterations, because there is no clear requirement for determining the ANN system. Consequently, the practical implementation of such a control method is constrained.

Model Predictive Control (MPC) encompasses a collection of controllers that rely on the system model and anticipated future references to compute optimal control signals. The fundamental principle of predictive control involves precalculating the control signal needed for the system based on prior knowledge of the upcoming input reference that is going to be utilized^14^, where the system can respond to the input reference by predicting its alterations, limiting the impacts of system delays. Following the introduction of the concepts of generalized predictive control strategy by^15^, numerous authors have applied this advanced technique in the past two decades for the control of induction motors. All predictive control methods rely on minimizing a cost function. In the case of MPC, this involves addressing a quadratic programming problem, particularly when incorporating physical constraints into the optimization process. Without constraints, it is possible to derive an analytical solution^16^. However, it is important to note that real-world systems inherently exhibit constraints, such as saturation values, frequency limitations, and temporal constraints on the actuators. A predictive controller’s sample time is typically greater than that of other kinds of controllers, limiting its application to fast response systems. To address this concern, certain studies disregard constraints, opting for the analytical solution, which yields satisfactory results. Furthermore, constraints can be incorporated after deriving the predictive control law^17^. Moreover, although the delay time in electric motor systems is typically minimal, there are instances where it becomes significant enough that compensating for it markedly enhances system behavior. This characteristic makes it applicable in precision-oriented applications. Within this context, algorithms for prediction expertly adjust for the regulated system’s delay time, as these algorithms inherently account for this aspect in their implementation. However, during the application process, model parameter uncertainty is widespread. Various factors, such as changes in induction motor temperature, and magnetic saturation in the stator winding during operation, lead to a mismatch in stator resistance and inductance parameters^18^. Additionally, in electric vehicle applications employing the induction motor drive, significant variations in load torque frequently occur. The drive speed servo system must ensure optimal response performance, particularly in the presence of substantial external load disturbances. Consequently, both low system model accuracy and parameter mismatches, along with external load disturbances, directly impact the control precision of the system. Hence, challenges primarily revolve around model parameter mismatch and changes in load characteristic parameters, prompting an increasing focus from researchers on robust control in the context of MPC. In reference^19^, a motor-predictive torque control technique was put forth. Within the outer speed loop, a load disturbance observer (DOB) is employed to estimate the overall disturbance, leading to a quicker torque reference. Simultaneously, in the inner current loop, real-time observation of parameters is achieved through stator flux and torque observers. In^20^, a deadbeat current predictive control approach is introduced, aiming to suppress all parameter disturbances. Although successful in diminishing parameter sensitivity, this method necessitates the design of two observers, thereby introducing additional complexity.

The primary purpose of this paper is to improve the control and effectiveness of the performance of electric vehicle powertrains by introducing a new robust model predictive control approach. Regarding the regulation of speed, the robustness of the system is enhanced by altering the cost function of the conventional model predictive control, a method that relies on Taylor series expansion. The new law of the MPC incorporates integral action, consequently, under the assumption of a stable closed-loop system, the suggested controller successfully eradicates steady-state error, even when confronted with unknown perturbations and parameter variations. Additionally, the suggested model predictive direct torque control law, grounded in a cost function, serves as a substitute for conventional hysteresis controllers and switching tables. It involves selecting the voltage space vector sequence in the upcoming sampling cycle that minimizes torque and flux magnitude errors. The incorporation of a cost function provides the controller with a significant amount of flexibility, allowing for the integration of system nonlinearities and limitations into the optimization process. The primary contributions of this article can be outlined as follows:

- We introduce a novel robust predictive control strategy that substitutes traditional switching tables and hysteresis controllers with a predictive block utilizing an optimization algorithm. This innovation markedly reduces high torque and flux ripples, addressing a significant limitation in traditional DTC methods.
- By modifying the cost function in conventional MPC and integrating the new law into the regulating speed loop, the proposed controller not only eliminates errors but also enhances disturbance rejection capabilities and resilience to parameter variations. This ensures closed-loop system stability and overall robustness.
- Implementing the proposed control system results in a consistent switching frequency, thereby significantly enhancing the direct torque control. Consequently, this technique is more suitable for implementation in electric vehicle systems.

The rest of this article follows this structure: Sections "Electric Vehicle Dynamic Model" and "Induction Motor Model" are dedicated to the mathematical models for both the electric vehicle and the induction motor, respectively. Section "Fundamental Theoretical Formulation" provides an overview of the fundamental theoretical formulation of classic DTC. Section "Proposed Robust Model Predictive Direct Torque Control Scheme" describes the formulation and development of the suggested robust model predictive DTC scheme. Sections "Simulation Results" and "Experimental Validation Using The OPAL-RT Platform" display the outcomes of simulations, outline the experimental arrangement, and present the results obtained from the experiments. Finally, Section 8 concludes the paper.

## Electric vehicle dynamic model

**Figure 1:**
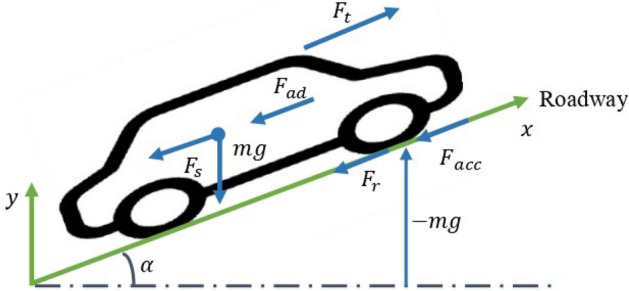
Forces exerted on an electric vehicle.

The initial stage of vehicle modeling involves identifying the various forces that impact the vehicle’s dynamics. These forces include aerodynamic drag force ($F_{ad}$), rolling resistance force ($F_{r}$), sloping force ($F_{s}$), friction force in bearings ($F_{bear}$), and acceleration force ($F_{acc}$), as illustrated in Fig. 1^21^:

### Aerodynamic force

The friction of the vehicle’s body as it moves through the air generates aerodynamic force, as well as the velocity of the wind, it depends on the vehicle’s frontal surface area, its design, and the wind’s direction. The drag force is stated below^22^:

1 $$\begin{aligned} F_{ad}=\frac{1}{2}\rho A_fC_d(v+v_w)^2 \end{aligned}$$

With $\rho$ stands for the density of air, $A_f$ denotes the frontal area of the vehicle,$C_d$ represents the aerodynamic drag coefficient, *v* indicates the longitudinal speed of the vehicle, and $v_w$ is the wind speed.

### Rolling resistance force

When the wheels of the vehicle turn, a section of each tire is consistently pushed down and then released as it moves away from the surface. These actions of the tires consume energy, what is known as rolling resistance, which acts against the vehicle’s movement. The force of rolling resistance is denoted by:

2 $$\begin{aligned} F_{r}=m_vgf_rcos(\alpha ) \end{aligned}$$

Where $m_v$ indicates the vehicle’s mass, *g* stands for gravity acceleration, $\alpha$ represents the road inclination angle and $f_r$ denotes the rolling resistance coefficient.

### Sloping force

The sloping force signifies the fraction of tractive force necessary for propelling a vehicle uphill at a particular angle ($\alpha$). This force is generated as a result of the vehicle’s mass in motion when ascending or descending a hill. The expression for the sloping force is as follows:

3 $$\begin{aligned} F_{s}=m_vgsin(\alpha ) \end{aligned}$$

### Friction force in bearings

The frictional force in the vehicle’s bearings is expressed as follows:

4 $$\begin{aligned} F_{bear}=\frac{K_b v_w}{r} \end{aligned}$$

With $K_b$ bearing friction coefficient, *r* is the wheel radius and $v_w$ vehicle wheel velocity of rotation.

### Acceleration force

The amount of force necessary for opposing the mass of the vehicle’s inertia to accelerate, and is defined in the following manner:

5 $$\begin{aligned} F_{acc}=m_v\frac{dv}{dt} \end{aligned}$$

### Tractive force

**Figure 2:**
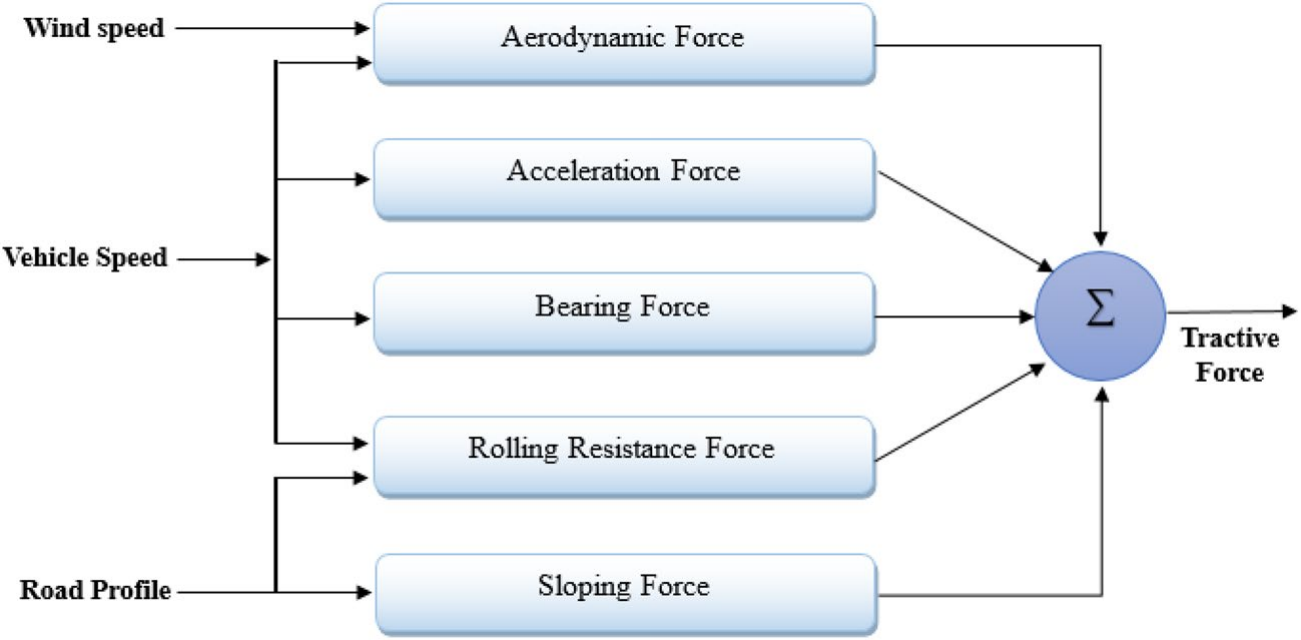
Electric vehicle’s tractive force.

The force conveyed to those wheels via a transmission mechanism to set the vehicle in motion is referred to as the entire tractive force. As indicated in Fig. 2, it is the sum of aerodynamic force, the force of rolling resistance, sloping force, friction force in bearings, and acceleration force. The tractive force can be expressed as follows^9^:

6 $$\begin{aligned} F_t=F_{ad}+F_{r}+F_{s}+F_{bear}+F_{acc} \end{aligned}$$

Then, the tractive torque produced by the vehicle’s propulsion motor equals:

7 $$\begin{aligned} T_m=\frac{F_t*r}{G}=\frac{1}{ G}\big [F_{ad}+F_{r}+F_{s}+F_{bear}+F_{acc}\big ]r \end{aligned}$$

Where *G* represents the gear ratio.

## Induction motor model

Mathematical modeling of electrical motors is essential to analyze the dynamic behavior of these devices under different operating conditions. The induction motor’s mathematical modeling within the stator-fixed reference frame ($\alpha , \beta$) used in the following analysis is stated as^23^ :

8 $$\begin{aligned} \frac{d}{dt}\begin{bmatrix} i_{s\alpha }\\ i_{s\beta }\\ \psi _{s\alpha }\\ \psi _{s\beta } \end{bmatrix}=\begin{bmatrix} -\frac{1}{\sigma }\Big (\frac{1}{\tau _s}+\frac{1}{\tau _r}\Big )&{}\quad -\omega _r&{}\quad \frac{1}{\sigma L_s \tau _r}&{}\quad \frac{\omega _r}{\sigma L_s}\\ \omega _r &{} -\frac{1}{\sigma }\Big (\frac{1}{\tau _s}+\frac{1}{\tau _r}\Big ) &{}\quad \frac{-\omega _r}{\sigma L_s}&{}\quad \frac{1}{\sigma L_s \tau _r}\\ -R_s &{}\quad 0 &{}\quad 0 &{}\quad 0\\ 0 &{}\quad -R_s &{}\quad 0 &{}\quad 0 \end{bmatrix}\begin{bmatrix} i_{s\alpha }\\ i_{s\beta }\\ \psi _{s\alpha }\\ \psi _{s\beta } \end{bmatrix}+\begin{bmatrix} \frac{1}{\sigma L_s} &{}\quad 0\\ 0 &{}\quad \frac{1}{\sigma L_s}\\ 1 &{}\quad 0\\ 0 &{}\quad 1 \end{bmatrix} \begin{bmatrix} v_{s\alpha }\\ v_{s\beta } \end{bmatrix} \end{aligned}$$

With $$\begin{array}{ccc} \tau _s=\frac{L_s}{R_s};&\quad \tau _r=\frac{L_r}{R_r};&\quad \sigma =1-\frac{L^2_m}{L_sL_r} \end{array}$$

The expression of the electromagnetic torque is as follows:

9 $$\begin{aligned} T_{em}=p(\psi _{s\alpha } i_{s\beta }-\psi _{s\beta } i_{s\alpha }) \end{aligned}$$

The mechanical equation is given as follows:

10 $$\begin{aligned} \frac{d\omega _r}{dt}= \frac{1}{J}(T_{em}-T_L-f\omega _r) \end{aligned}$$

Where ($i_{s\alpha },i_{s\beta }), (v_{s\alpha },v_{s\beta })$ and $(\psi _{s\alpha },\psi _{s\beta })$ represent the $\alpha , \beta$ components current, stator voltage, and flux, respectively. $R_s$ and $R_r$ denote respectively the stator and rotor resistances, $L_s$ is the stator inductance, $L_r$ is the rotor inductance, $L_m$ is the magnetizing inductance, $\omega _r$ represent the mechanical rotor speed, $\sigma$ represents the coefficient of leakage, $\tau _s$ and $\tau _r$ are the electrical time constants of the stator and rotor, respectively, $T_L$ the load torque, *J* the inertia moment, *p* represents the number of pole pairs and *f* the coefficient of friction.

## Fundamental theoretical formulation

The fundamental concept of direct torque control relies on applying a control sequence directly to the voltage inverter switches located before the machine. The selection of this sequence is determined using a switching table, alongside two hysteresis regulators, which serve to manage and regulate the electromagnetic torque and the machine’s flux^24^. The electromagnetic torque is managed through a three-level hysteresis regulator, whereas the stator flux is regulated using a two-level hysteresis regulator. The results from these regulators, along with the data of the flux vector, are utilized to establish the switching table^25^. In the context of DTC, ensuring the precise estimation of electromagnetic torque and stator flux is crucial for achieving satisfactory system performance, as a result, multiple parameters need to be ascertained. The current is directly determined, whereas the voltage is contingent upon the switching state ($S_a, S_b,$ and $S_c$) determined by the switching table. The transformation of these parameters into coordinates $(\alpha , \beta )$ is conducted via the Concordia transformation, as follows^5^ :

11 $$\begin{aligned} \begin{bmatrix} x_{s\alpha }\\ x_{s\beta }\\ \end{bmatrix}= \sqrt{\frac{2}{3}} \begin{bmatrix} 1&{}\quad -\frac{1}{2}&{}\quad -\frac{1}{2}\\ 0&{}\quad \frac{\sqrt{3}}{2}&{}\quad -\frac{\sqrt{3}}{2} \end{bmatrix} \begin{bmatrix} x_{sa}\\ x_{sb}\\ x_{sc} \end{bmatrix} \end{aligned}$$

The following equations are used to estimate the flux in the stator (${\hat{\psi }}_s$) and the electromagnetic torque (${\hat{T}}_{em}$)^24^:

12 $$\begin{aligned}{} & {} \hat{\psi }_s= \sqrt{\hat{\psi }_{s\alpha }^2+\hat{\psi }_{s\beta }^2} \end{aligned}$$

13 $$\begin{aligned}{} & {} \hat{T}_{em}=p(\hat{\psi }_{s\alpha } i_{s\beta }-\hat{\psi }_{s\beta } i_{s\alpha }) \end{aligned}$$

The following are the elements of stator flux:

14 $$\begin{aligned} \left\{ \begin{array}{c} \hat{\psi }_{s\alpha }=\int (v_{s\alpha }-R_si_{s\alpha })\,dt\\ \hat{\psi }_{s\beta }=\int (v_{s\beta }-R_si_{s\beta })\,dt\ \end{array} \right. \end{aligned}$$

The formula that follows is employed to determine the stator flux position:

15 $$\begin{aligned} \theta =\tan ^{-1}\frac{\hat{\psi }_{s\beta }}{\hat{\psi }_{s\alpha }} \end{aligned}$$

Subsequently, the compared values of the estimated flux ($\hat{\psi }_s$) and electromagnetic torque ($\hat{T}_{em}$) with their corresponding reference ($\hat{\psi }_{ref}$ and $\hat{T}_{ref}$) serve as hysteresis comparators’ inputs. Choosing the suitable voltage vector is determined by the control table. The flux sector identifier and the outputs of each of the hysteresis comparators are fed into the table^26^. Although the DTC is recognized for its straightforwardness, resilience, and swiftness, it also comes with notable drawbacks. Utilizing hysteresis controllers leads to significant ripples in flux and electromagnetic torque, as a result, it leads to current distortions and variable switching frequencies, which have the potential to compromise the quality of the output power and adversely affect the overall performance of the motor. Furthermore, the commonly employed approach in the DTC for speed control involves the use of a PI controller to eliminate static error and minimize response time, however, The speed typically exhibits an initial overshoot during startup and it’s influenced by the machine’s parameters^5,27^. Within this framework, to address the drawbacks of the traditional DTC a new control scheme is proposed and will be elaborated in the following section.

## Proposed robust model predictive direct torque control scheme

**Figure 3:**
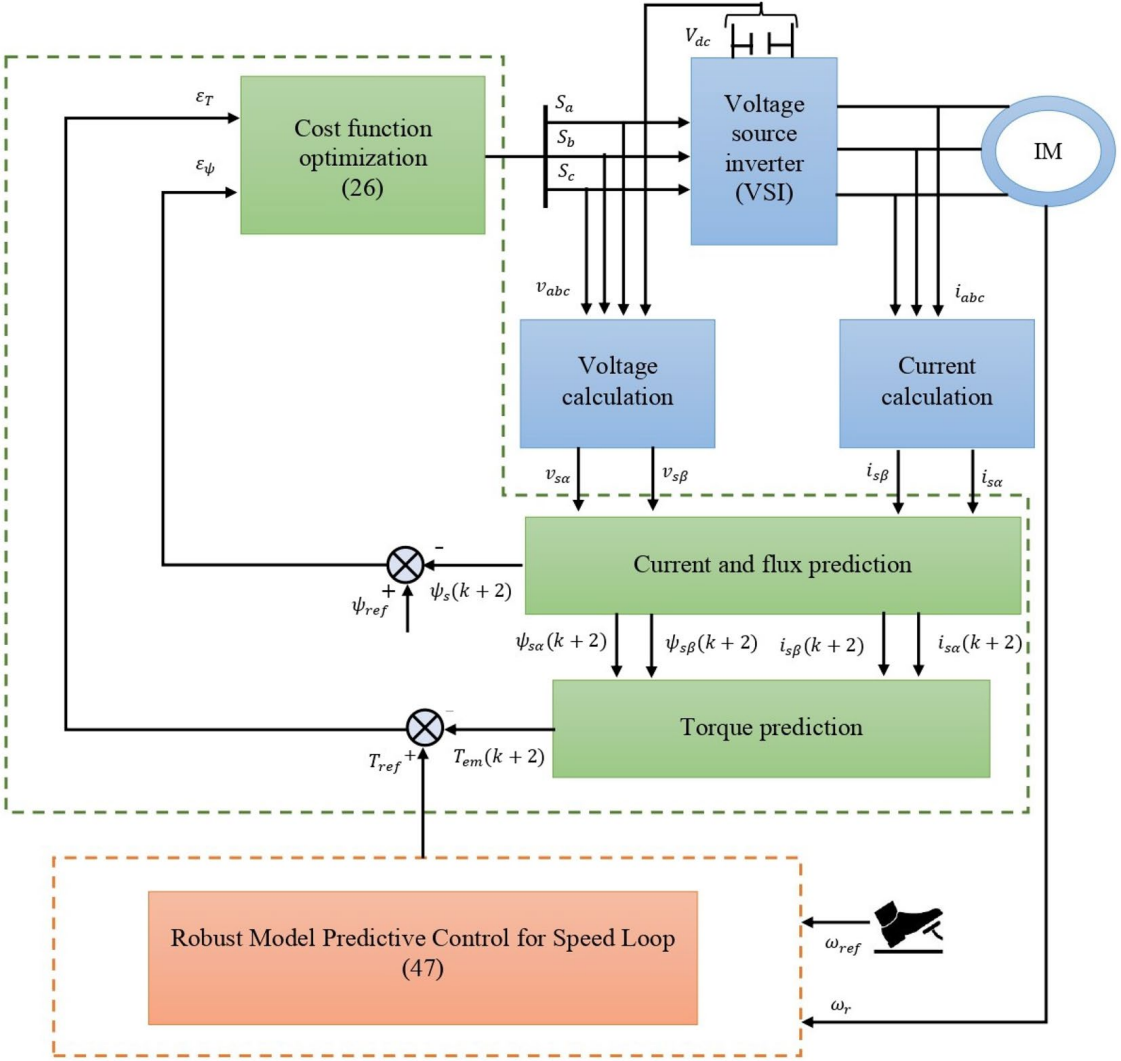
Diagram illustrating the proposed robust model predictive direct torque control.

This section introduces a new approach centered on robust predictive control, optimizing a newly defined cost function to enhance the traditional DTC strategy. The predictive block replaces the conventional switching table and hysteresis controllers. Additionally, the PI controller is substituted by a robust predictive speed controller, providing the reference of torque to the predictive block, as illustrated in Fig 3.

### Prediction algorithm for torque control

Within this subsection, the model predictive DTC block is implemented to regulate flux and torque in the induction motor. The block’s function is to anticipate the controlled variables for all eight potential space vectors representing stator voltage, denoted as $\textbf{V}_{i=0:7}$. Predictions involve the utilization of a specified cost function, the outcomes of the predictions aim to minimize it for upcoming control actions, achieved by choosing the appropriate voltage vector^28^. The execution of the MPDTC algorithm consists of two distinct phases. The first phase involves forecasting the controlled variables, while the second phase entails choosing the voltage vector for the upcoming sampling interval. A cost function is formulated to identify the most suitable voltage vector for use in the subsequent sampling period. The equation used for calculating stator current prediction is as follows^29^:

16 $$\begin{aligned}{} & {} i_{s\alpha } (k+1)=\Big (1+\frac{T_s}{\tau _\sigma }\Big ) i_{s\alpha }(k)+\frac{T_s}{\tau _\sigma + T_s} \Big (\frac{1}{R_\sigma }\frac{k_r}{\tau _r-jk_r \omega _r}\Big )\psi _{s\alpha } (k)+v_{s\alpha }(k) \end{aligned}$$

17 $$\begin{aligned}{} & {} i_{s\beta } (k+1)=\Big (1+\frac{T_s}{\tau _\sigma }\Big ) i_{s\beta }(k)+\frac{T_s}{\tau _\sigma + T_s} \Big (\frac{1}{R_\sigma }\frac{k_r}{\tau _r-jk_r \omega _r}\Big )\psi _{s\beta } (k)+v_{s\beta }(k) \end{aligned}$$

where $T_s$ represents the sampling time, and: $$\begin{array}{ccc} k_r=\frac{L_m}{L_r};&R_{\sigma }=R_s+R_r k_r^2;&\tau _{\sigma }=\sigma \frac{L_s}{R_{\sigma }}. \end{array}$$

The prediction of stator flux in the ($\alpha , \beta$) frame is represented through (18)–(20) as demonstrated below:

18 $$\begin{aligned}{} & {} \psi _{s\alpha } (k+1)= \psi _{s\alpha } (k)+T_s(v_{s\alpha } - R_s i_{s\alpha }) \end{aligned}$$

19 $$\begin{aligned}{} & {} \psi _{s\beta } (k+1)= \psi _{s\beta }(k)+T_s(v_{s\beta } - R_s i_{s\beta }) \end{aligned}$$

20 $$\begin{aligned}{} & {} \psi _s (k+1)= \sqrt{(\psi _{s\alpha } (k+1))^2+(\psi _{s\beta } (k+1))^2} \end{aligned}$$

The electromagnetic torque is calculated based on the predicted current and flux in the following manner:

21 $$\begin{aligned} T_{em}(k+1)=\frac{3}{2}p(\psi _{s\alpha } (k+1) i_{s\beta } (k+1)-\psi _{s\beta } (k+1) i_{s\alpha }(k+1)) \end{aligned}$$

The optimum flux and torque are obtained by optimizing a cost function $\Upsilon$, expressed as follows:

22 $$\begin{aligned} \Upsilon = |T_{ref}- T_{em}(k+1)|+ \Lambda |\psi _{ref}- \psi _s(k+1)| \end{aligned}$$

In this context, $T_{ref}$ and $T_{em}(k+1)$ denote the reference and the predicted torque at $\textbf{V}_{i=0:7}$, respectively. Similarly, $\psi _{ref}$ and $\psi _s(k+1)$ represent the flux reference and the predicted flux. A constant denoted as $\Lambda$, which permits the adjustment of the significance of flux error relative to torque error, set to 20 in this case. At moment $(k + 1)$, the voltage vector specified will be implemented through a one-step delay, since the sampling time is minute, this can compromise the system’s performance. As a result, compensating for the delay time is essential to enhance system performance, by diminishing torque ripples and mitigating the impact of measurement noise^30^. Hence, the forecast for two steps ahead $(k + 2)$ will be taken into account. The stator currents prediction at time $(k + 2)$ can be described by:

23 $$\begin{aligned}{} & {} i_{s\alpha } (k+2)=\Big (1+\frac{T_s}{\tau _\sigma }\Big )i_{s\alpha }(k+1)+\frac{T_s}{\tau _\sigma + T_s} \Big (\frac{1}{R_\sigma }\frac{k_r}{\tau _r-jk_r \omega _r}\Big )\psi _{s\alpha } (k+1)+v_{s\alpha }(k+1) \end{aligned}$$

24 $$\begin{aligned}{} & {} i_{s\beta } (k+2)=\Big (1+\frac{T_s}{\tau _\sigma }\Big )i_{s\beta }(k+1)+\frac{T_s}{\tau _\sigma + T_s} \Big (\frac{1}{R_\sigma }\frac{k_r}{\tau _r-jk_r \omega _r}\Big )\psi _{s\beta } (k+1)+v_{s\beta }(k+1) \end{aligned}$$

The cost function (22) can be expanded by introducing a constraint on the current magnitude to prevent overcurrent, this constraint is defined as follows:

25 $$\begin{aligned} \hat{f}\big (i_{s\alpha } (k+2), i_{s\beta } (k+2)\big )= \begin{array}{c} \infty \begin{array}{c} if |i_{s\alpha } (k+2)|> i_{max}\\ or |i_{s\beta } (k+2)|> i_{max} \end{array} \end{array}\quad and \quad \begin{array}{c} 0 \begin{array}{c} if |i_{s\alpha } (k+2)|\le i_{max}\\ or |i_{s\beta } (k+2)|\le I_{max} \end{array} \end{array} \end{aligned}$$

The overall cost function $\Upsilon$ of the MPDTC with compensation for computational time delays is expressed as:

26 $$\begin{aligned} \Upsilon = |T_{ref}-T_{em}(k+2)|+ \Lambda |\psi _{ref}- \psi _s(k+2)|+ \hat{f}\Big (i_{s\alpha } (k+2), i_{s\beta } (k+2)\Big ) \end{aligned}$$

**Figure 4:**
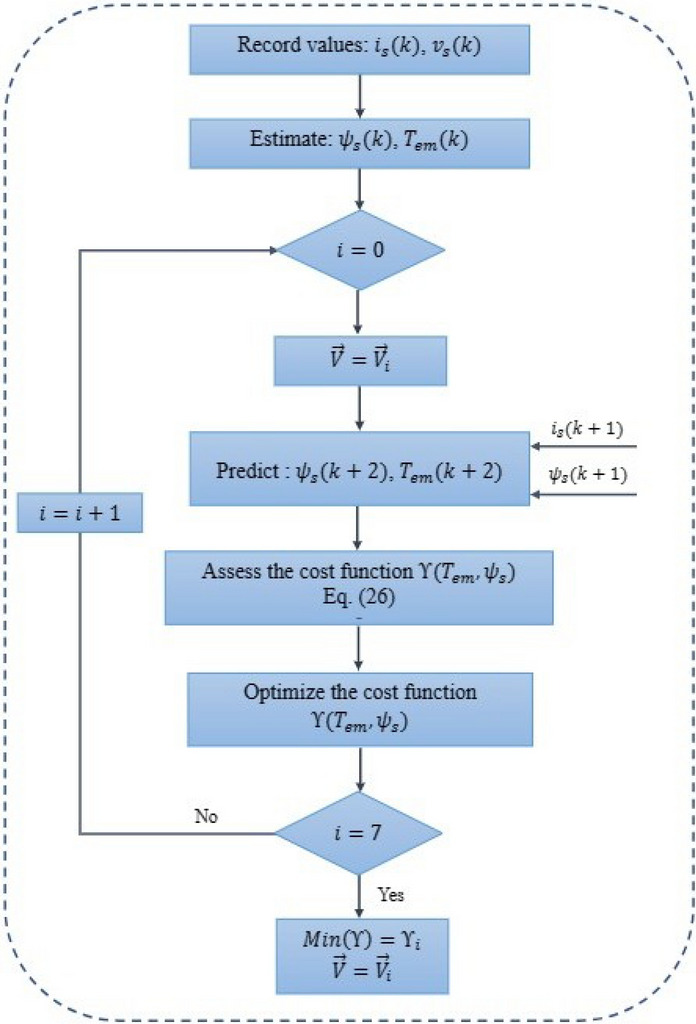
The flowchart of the prediction algorithm for torque control.

At each voltage space vector, the cost function $\Upsilon$ is assessed, with only one vector value minimizing the cost function, Fig. 4 depicts the prediction algorithm’s flowchart. The specific scenario of a three-phase inverter leads to the availability of eight voltage space vectors, itemized in Table 1.

**Table 1:** States of the inverter and their corresponding space vectors.

$S_a,S_b,S_c$	${\bf {\overrightarrow V}}_i$
0,0,0	${\bf {\overrightarrow V}}_0=0$
1,0,0	${\bf {\overrightarrow V}}_1=\sqrt{\frac{2}{3}}V_{dc}$
1,1,0	${\bf {\overrightarrow V}}_2=\sqrt{\frac{2}{3}}V_{dc} e^{j\pi /3}$
0,1,0	${\bf {\overrightarrow V}}_3=\sqrt{\frac{2}{3}}V_{dc} e^{j2\pi /3}$
0,1,1	${\bf {\overrightarrow V}}_4=-\sqrt{\frac{2}{3}}V_{dc}$
0,0,1	${\bf {\overrightarrow V}}_5=\sqrt{\frac{2}{3}}V_{dc} e^{j4\pi /3}$
1,0,1	${\bf {\overrightarrow V}}_6=\sqrt{\frac{2}{3}}V_{dc} e^{j5\pi /3}$
1,1,1	${\bf {\overrightarrow V}}_7=0$

### Robust model predictive speed controller

In this part, the mathematical model and the concept of the robust nonlinear model predictive approach, incorporating the newly proposed robust law are presented. The fundamental objective of the classic predictive controller is to identify the optimal input that ensures the predicted plant output $y(t+\tau )$ perfectly follows a future reference trajectory $y_{ref}(t+\tau )$, taking into account potential disturbances^31^. This requirement corresponds to minimizing the cost function as defined by^32^ :

27 $$\begin{aligned} \xi =\frac{1}{2}\int _{0}^{T} [y_{ref}(t+\tau )-y(t+\tau ))^T(y_{ref}(t+\tau )-y(t+\tau )] \,d\tau \end{aligned}$$

To address the optimization problem (27), the anticipated output and the reference trajectory are both expanded into a Taylor series of order $\rho _i$ employing the Lie derivative, $\rho _i$ denotes the relative degree of any given output. The drawback of the classic PC mentioned earlier is its reliance on having prior knowledge of the disturbance. To bolster the predictive control’s robustness, a novel nonlinear predictive control law has been suggested, we have incorporated an integral action into the controller when designing it without considering the disturbance. The inclusion of integral action within the controller can be achieved by adopting a novel cost function, which is presented as follows:

28 $$\begin{aligned} \xi =\frac{1}{2}\int _{0}^{T}\Pi (t+\tau )^T\Pi (t+\tau ) \,d\tau \end{aligned}$$

With

29 $$\begin{aligned} \Pi (t)= \int _{0}^{t}[y_{ref}(\tau )-y(\tau )]\,d\tau \end{aligned}$$

To address the nonlinear optimization problem (28), the anticipated term is extended through a Taylor series expansion, up to the order of $(\rho _i+1)$:

30 $$\begin{aligned} \Pi (t+\tau )=\Pi _i(t)+\sum _{n=1}^{\rho _i+1} \frac{\tau ^n}{n!}\Pi _i^{(n)}(t) \end{aligned}$$

Lie derivation of the function *h*(*x*) is represented by:

31 $$\begin{aligned} \left\{ \begin{array}{c} L_A h_i(x)=\frac{\delta h_j}{\delta x}A(x) \\ L^n_Ah_i(x)=L_A(L^{n-1}_Ah_i(x))\\ L_BL_Ah_i(x)=\frac{\delta L_A h_i}{\delta x}B_u(x) \end{array} \right. \end{aligned}$$

Substituting Eq. (29) into (30), and invoking (31) results in:

32 $$\begin{aligned} \Pi (t+\tau )=\Pi _i(t)+\sum _{n=1}^{\rho _i+1} \frac{\tau ^n}{n!}[y_{ref}^{(n-1)}(t)-L_A^{(n-1)}h_i(x)]+\frac{\tau ^{(\rho _i+1)}}{(r_i+1)!}L_BL_A^{(\rho _i-1)}h_i(x)u(t) \end{aligned}$$

When all output systems have an equal relative degree, it is feasible to write (32) as follows:

33 $$\begin{aligned} \Pi (t+\tau )= \Sigma (\tau )[E(t)-\Psi (u)] \end{aligned}$$

Where

34 $$\begin{aligned} \Sigma (\tau )=\begin{bmatrix} \Sigma _1(\tau )\\ \Sigma _2(\tau )\\ \Sigma _3(\tau )\\ .\\ \Sigma _{(\rho _i+1)}(\tau ) \end{bmatrix}; E(t)=\begin{bmatrix} \int _{0}^{t}[y_{ref}(\tau )-y(\tau )] \,d\tau \\ y_{ref}(\tau )-y(\tau )\\ y_{ref}^{(1)}(\tau )-y^{(1)}(\tau )\\ .\\ y_{ref}^{(\rho _i)}(\tau )-y^{(\rho _i)}(\tau ) \end{bmatrix}; \Psi (u)=\begin{bmatrix} 0_{m*n}\\ 0_{m*n}\\ 0_{m*n}\\ .\\ M_u(x) \end{bmatrix}u(t) \end{aligned}$$

With

35 $$\begin{aligned} \left\{ \begin{array}{c} \Sigma _n(\tau )=\frac{\tau ^{(n)}}{(n)!}I_d\\ M_u(x)=\begin{bmatrix} M_1(x) &{} M_2(x) &{}... &{} M_m(x) \end{bmatrix}^T \end{array} \right. \end{aligned}$$

Substituting Eq. (33) into Eq. (28), the resulting expression for the new cost function will be :

36 $$\begin{aligned} \xi =\frac{1}{2}\begin{bmatrix}E(t)-\Psi (u) \end{bmatrix}^T \hat{\Lambda }(\tau ) \begin{bmatrix}E(t)-\Psi (u) \end{bmatrix} \end{aligned}$$

Where

37 $$\begin{aligned} \hat{\Lambda }(\tau )=\Sigma (\tau )^T\Sigma (\tau ) \end{aligned}$$

The stipulated prerequisite for achieving optimal control is characterized by:

38 $$\begin{aligned} \frac{d\xi }{du}=0 \end{aligned}$$

#### Implementing robust model predictive control for IM

The design of the synthesis of the speed controller in this research relies on a robust predictive control approach. Indeed, the new cost function (28) has been implemented to ensure the control’s high efficiency and resilience, where the load torque is ignored since it is seen as an unknown perturbation. The representation in matrix form for the mechanical dynamics is in the following manner:

39 $$\begin{aligned} \left\{ \begin{array}{c} \dot{x}(t)=A(x)+B(x)u(t) \\ y(t)=h(x) \end{array} \right. \end{aligned}$$

Where $x(t)=\omega _r$, $u(t)=T_{em}$, and $y(t)=\omega _r$ are the vectors representing the states, inputs, and outputs, respectively. A(x) and B(x) are defined by

40 $$\begin{aligned} \left\{ \begin{array}{c} A(x)=-\frac{f}{J}\omega _r\\ B(x)=\frac{1}{J} \end{array} \right. \end{aligned}$$

Because the relative degree of the output y(t) is $\rho _\omega =1$, this indicates that:

41 $$\begin{aligned} \left\{ \begin{array}{c} y(t)=h(x)\\ \dot{y}(t)=L_Ah(x)+M(x)u(t) \end{array} \right. \end{aligned}$$

With $$M(x)=\frac{f}{J^2}$$

The anticipated term $\Pi (t+\tau )$ is subjected to expansion through a Taylor series of $(\rho + 1){th}$ order as follow:

42 $$\begin{aligned} \Pi (t+\tau )=\Pi (t)+\tau [\omega _{ref}(\tau )-\omega _r(\tau )]+\frac{\tau ^2}{2} \begin{bmatrix} \dot{\omega }_{ref}(t)-L_Ah(x)-M(x)u(t) \end{bmatrix} \end{aligned}$$

Combining (41) and (42) yields:

43 $$\begin{aligned} \Pi (t+\tau )= \Sigma (\tau )[E(t)-\Psi (u)] \end{aligned}$$

Where

44 $$\begin{aligned} \Sigma (\tau )=\begin{bmatrix} 1&\tau&\frac{\tau ^2}{2} \end{bmatrix}; E(t)=\begin{bmatrix} \int _{0}^{t}[\omega _{ref}(\tau )-\omega _r(\tau )] \,d\tau \\ \omega _{ref}(\tau )-\omega _r(\tau )\\ \dot{\omega }_{ref}(t)-L_Ah(x) \end{bmatrix}; \Psi (u)=\begin{bmatrix} 0\\ 0\\ M(x) \end{bmatrix}u(t) \end{aligned}$$

The cost function obtained by substituting (43) into (28)is as follows

45 $$\begin{aligned} \xi =\frac{1}{2} \begin{bmatrix} \begin{bmatrix} \Pi (t)\\ \omega _{ref}(\tau )-\omega _r(\tau )\\ \dot{\omega }_{ref}(t)-L_Ah(x) \end{bmatrix}- \begin{bmatrix} 0\\ 0\\ M(x) \end{bmatrix} u(t) \end{bmatrix}^T \hat{\Lambda }(\tau ) \begin{bmatrix} \begin{bmatrix} \Pi (t)\\ \omega _{ref}(\tau )-\omega _r(\tau )\\ \dot{\omega }_{ref}(t)-L_Ah(x) \end{bmatrix}- \begin{bmatrix} 0\\ 0\\ M(x) \end{bmatrix} u(t) \end{bmatrix} \end{aligned}$$

Where

46 $$\begin{aligned} \hat{\Lambda }(\tau )=\begin{bmatrix} 1&\tau&\frac{\tau ^2}{2} \end{bmatrix}^T\begin{bmatrix} 1&\tau&\frac{\tau ^2}{2} \end{bmatrix} \end{aligned}$$

Respecting the condition (38), the optimal control can be achieved by:

47 $$\begin{aligned} u(t)=-M^{-1}\bigg [P_0\int _{0}^{t} e(\tau )\,d\tau +P_1e(t)+P_2\dot{e}(t)\bigg ] \end{aligned}$$

With

48 $$\begin{aligned} \begin{array}{ccc} P_0=\frac{2}{T_p};&P_1=\frac{2}{T_p^2};&P_2=1 \end{array} \end{aligned}$$

and

49 $$\begin{aligned} e(t)=\omega _{ref}(t)-\omega _r(t) \end{aligned}$$

#### System stability study

The examination of the stability of the tracking error at the origin allows us to tackle the overall stability of the closed-loop system. Achieving this can be accomplished by finding a characteristic expression which is obtained by inserting (47) into (41) :

**Table 2:** Parameters of the induction machine.

Descriptions	Parameters	Values
DC-link voltage	$V_{dc}$ (V)	400
Number of pole pairs	*p*	2
Mutual inductance	$L_m$ (H)	0.181
Stator inductance	$L_s$ (H)	0.194
Rotor inductance	$L_r$ (H)	0.194
Stator resistance	$R_s (\Omega )$	1.95
Rotor resistance	$R_r (\Omega )$	1.76
Inertia of IM	*J* (Kg m$^2$)	0.02

50 $$\begin{aligned} P_0\int _{0}^{t} e(\tau )\,d\tau +P_1e(t)+P_2\dot{e}(t)=0 \end{aligned}$$

Due to (50), the equation representing the characteristic polynomial is formulated as follows:

51 $$\begin{aligned} P_2s^2+P_1s+P_0=0 \end{aligned}$$

The solutions to (51) can be employed to obtain the stability condition, which is given below:

**Table 3:** Parameters of electric vehicle.

Descriptions	Parameters	Values
Vehicle mass	$m_v$ (kg)	1125
Frontal area	$A_f$ (m$^2$)	1.80
Rolling resistance coefficient	$f_r$	0.25
Drag coefficient	$C_d$	0.3

52 $$\begin{aligned} \left\{ \begin{array}{c} s_1=-\frac{1}{T_p}+\frac{1}{T_p}i\\ s_2=-\frac{1}{T_p}+\frac{1}{T_p}i \end{array} \right. \end{aligned}$$

Since each of the eigenvalues includes negative real parts, the closed-loop system demonstrates asymptotic stability. The tracking error dynamics are exclusively determined by the prediction time. A shorter prediction time is required for a quicker reaction.

**Figure 5:**
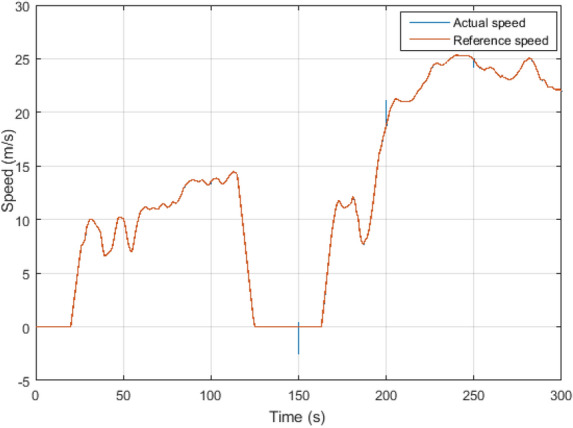
Response of actual and reference speeds under $T_L$ variation.

**Figure 6:**
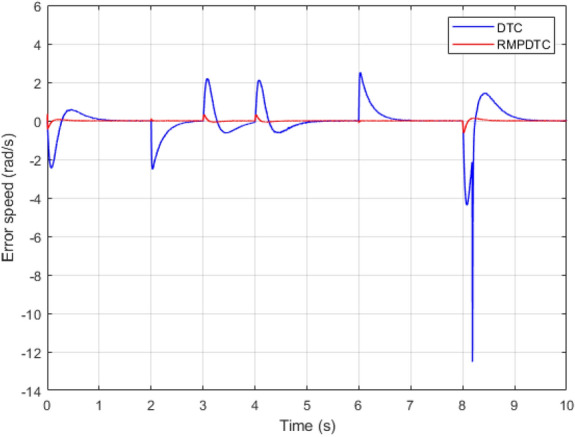
Response of the tracking speed error under $T_L$ variation.

**Figure 7:**
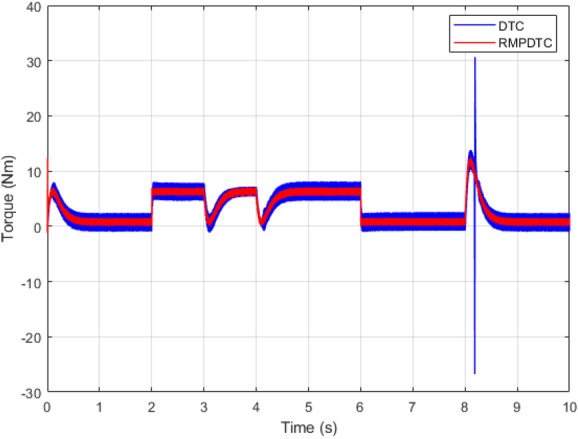
Response of electromagnetic torque under $T_L$ variation.

## Simulation results

In this section, we conducted simulations of the previously theoretically proposed control system employing Matlab/Simulink software. The suitability and efficiency of the proposed robust model predictive direct torque control (RMPDTC) are evaluated in contrast to the conventional direct torque control (DTC) approach. Performance graphs of the electric vehicle system’s induction motor were generated, and the simulation incorporated a prediction horizon of $T_p = 1$ ms, setting the reference flux at 0.8 Wb. The induction motor and EV parameters are detailed in Tables 2 and 3, respectively.

**Figure 8:**
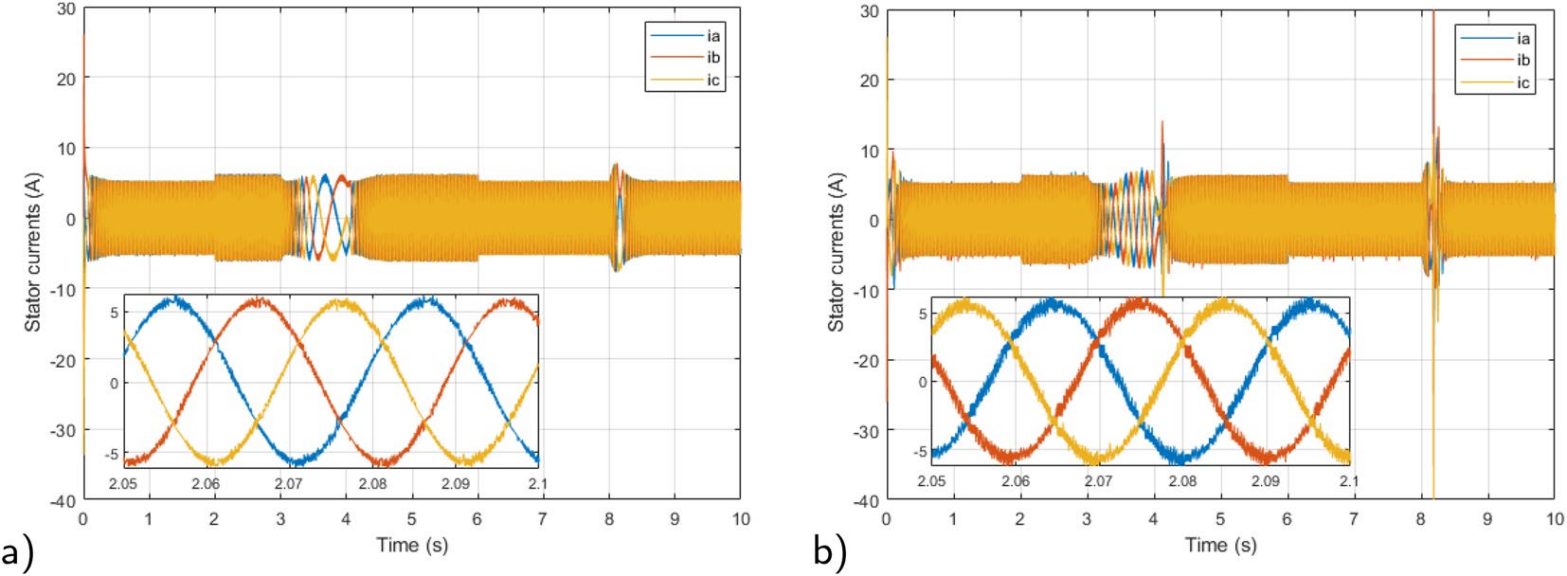
Three-phase stator currents under $T_L$ variation (**a**) Proposed RMPDTC (**b**) Classic DTC.

**Figure 9:**
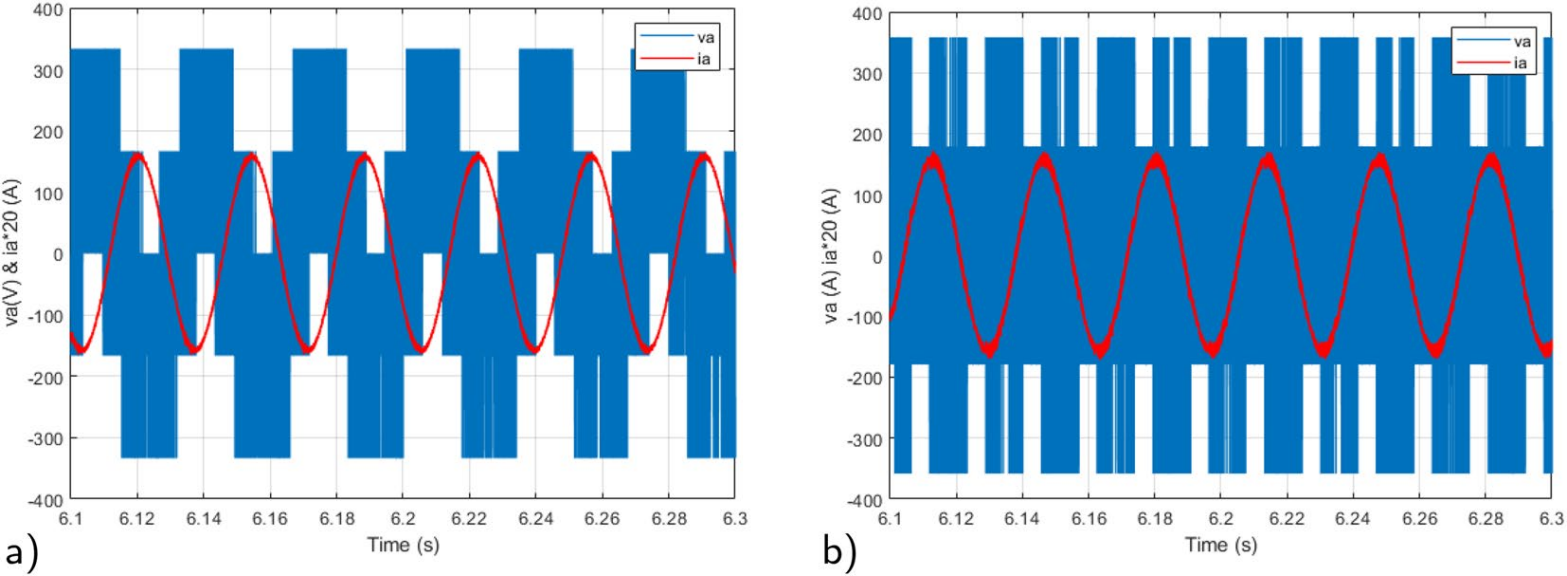
Rotor voltage and current under $T_L$ variation: (**a**) Proposed RMPDTC (**b**) Classic DTC.

**Figure 10:**
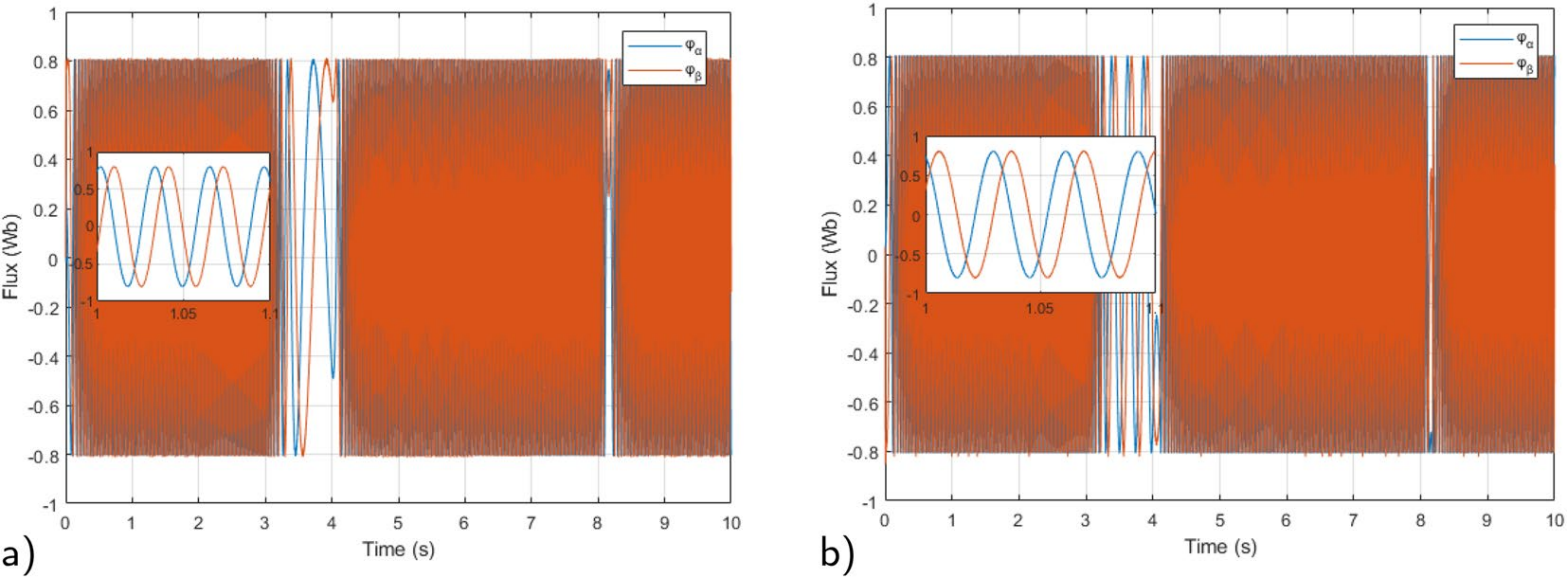
Stator Flux under $T_L$ variation (**a**) Proposed RMPDTC (**b**) Classic DTC.

**Figure 11:**
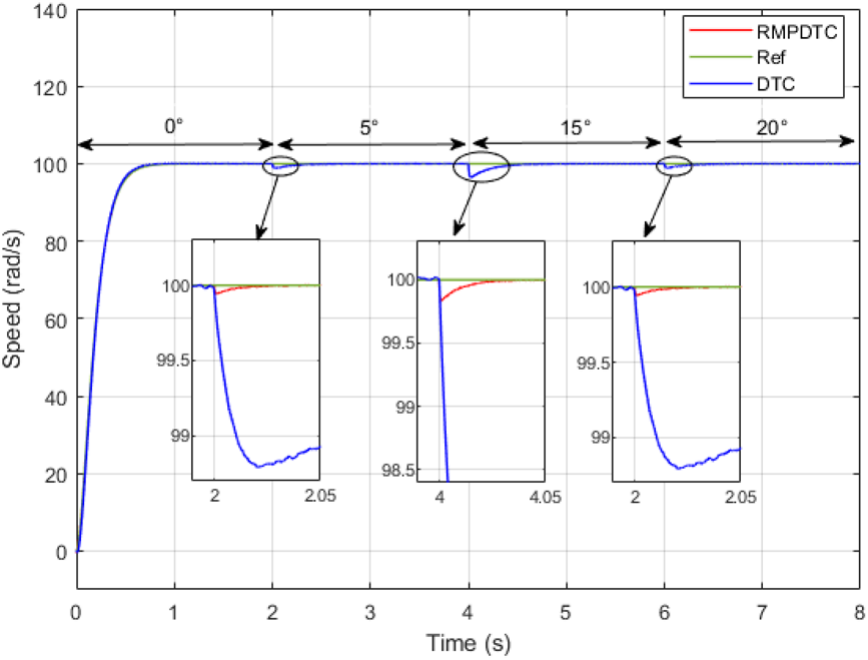
Response of actual and reference speeds under grade angle variation.

**Figure 12:**
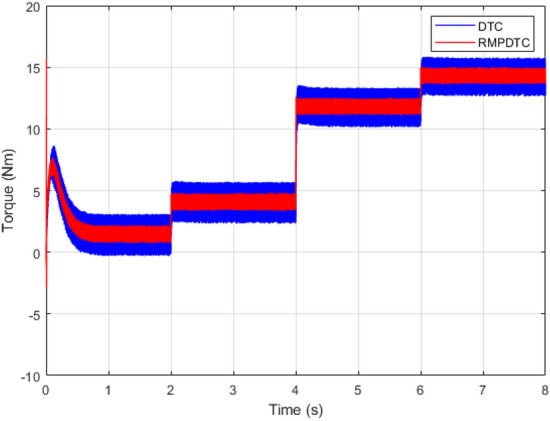
Response of electromagnetic torque under grade angle variation.

**Figure 13:**
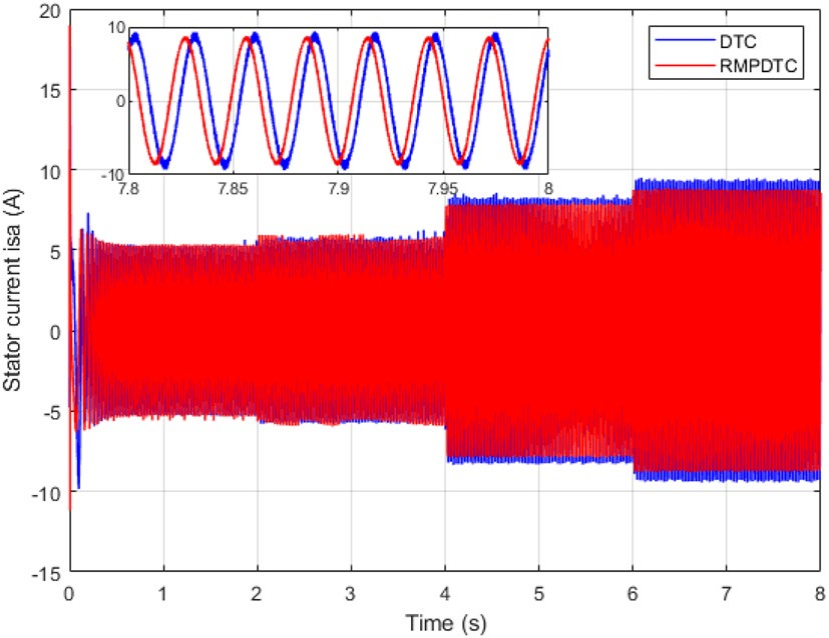
Stator phase current $(i_{sa})$ under grade angle variation.

### Robustness test under speed reverse and load torque variations

In this scenario, the motor speed transitions from 100 to − 100 rad/s at $t=4$ s, then from − 100 to 100 rad/s at $t=8$ s and a load torque of $T_L=7$ Nm is introduced at $t=2$ s. Figure 5a showcases the simulation outcomes for speed trajectory tracking in both DTC and the proposed RMPDTC, considering variations in load torque. We can notice that the classical DTC follows the reference speed, however, when subjected to load torque disturbance, it is evident that the classic controller is more adversely impacted than the proposed controller. In the proposed RMPDTC, the error is eliminated, and the controller effectively nullifies the impact of load torque. The speed response remains aligned with its reference point, demonstrating an extremely rapid response time.

Figure 6 displays the error response in tracking speed for each controller. The speed error plot illustrates successful outcomes with the suggested RMPDTC, demonstrating an overshooting or undershooting phenomenon of under 0.0004 in speed variations, even when faced with fluctuations in load torque. The employed approach enables rapid responsiveness, the absence of any noticeable steady-state inaccuracies, and minimum overshooting. In contrast to traditional DTC, the error grows with every shift in speed and load torque.

Figure 7 depicts graphs of electromagnetic torque for both controllers. The outcome underscores various significant enhancements when contrasted with the torque the traditional DTC generates, the implemented RMPDTC delivers a rapid torque response with minimal overshoot. The developed torque exhibits excellent responsiveness, characterized by negligible delays in its response. Moreover, substituting the hysteresis controllers and the switching table in DTC with a model predictive control block enables a substantial decrease in torque ripple across various operating conditions. An average overall enhancement of around $59\%$ is noted, indicating that the integration of the RMPDTC scheme plays a crucial role in significantly reducing the undesired torque ripple in DTC.

Figure 8 displays the stator current provided to the induction motor, furthermore, enlarged sections of the three-phase current are depicted. The stator current exhibits a sinusoidal pattern, and its magnitude correlates with the electromagnetic torque. The currents in the three phases are evenly distributed at intervals of $120^\circ$, following the typical configuration of a three-phase system. In the suggested RMPDTC system, there is a notable reduction in the current ripple, this outcome holds considerable importance since the substantial current ripple poses a significant drawback in traditional DTC, a drawback that RMPDTC can alleviate. Consequently, the peak current is reduced, leading to an enhancement in the quality of power delivered to the motor.

In Fig. 9 a zoom of stator voltage $v_a$ and current phase $i_a$ are illustrated, representing the voltage that supplies the IM. The primary distinction between the voltage waveforms in DTC and the suggested controller lies in the fact that, in the RMPDTC scheme, the voltage waveforms are produced at a consistent and unchanging switching frequency. Figure 10 displays the components of the stator flux along ($\alpha$,$\beta$) axes. The flux maintains a magnitude of around 0.8 Wb, regulated by the reference value for stator flux magnitude. The stator flux reference remained consistently set at a magnitude of 0.8 Wb throughout the simulation.

### Robustness test under grade angle variation

In the second scenario, as illustrated in Fig. 11, the objective is to maintain the velocity of the electric vehicle’s motor at 100 rad/s, even when facing variations in the grade angle. Initially, from $t=0$ s to $t=2$ s, the slope remains zero, indicating that the motor velocity precisely follows its designated reference, exhibiting a high accuracy in both controllers. Next, at $t=2$ s, $t=4$ s, and $t=6$ s, we implement slopes of $5^\circ , 15 ^\circ$, and $20^\circ$, respectively. The results indicate that the suggested method effectively keeps the motor speed consistent with the reference value, demonstrating robust tracking performance even when encountering changes in slope. The motor speed response also demonstrates the elimination of the chattering effect, leading to an absence of tracking error. This is in contrast to the DTC, where the error progressively increases with each alteration in slope. Figure 12 depicts the evolution of the electromagnetic torque for both controllers in response to changes in the grade angle. The RMPDTC shows a substantial reduction in torque ripples along with a rapid torque response when compared to the traditional DTC approach. Figure 13 illustrates the stator phase current $(i_{sa})$ for both traditional DTC and the suggested RMPDTC. Unlike the proposed method, in the classic DTC, there is a noticeable high-magnitude sinusoidal waveform, signifying a notable ripple level.

### Robustness test under parameter variations

To emphasize the efficiency of the suggested strategy, a comparative analysis is conducted between the designed RMPDTC and the conventional DTC to assess the resilience of the suggested control law in the face of parameter variations. In this scenario, the specified speed reference is sustained at 100 rad/s and the parameters subjected to testing are the rotor resistance ($R_r$) and mutual inductance ($L_m$). Testing involves augmenting the rotor resistance by $10 \%$ and the mutual inductance by $30\%$ from their rated values, this is achieved by modifying the machine parameters within the programming code of the current controller.

**Figure 14:**
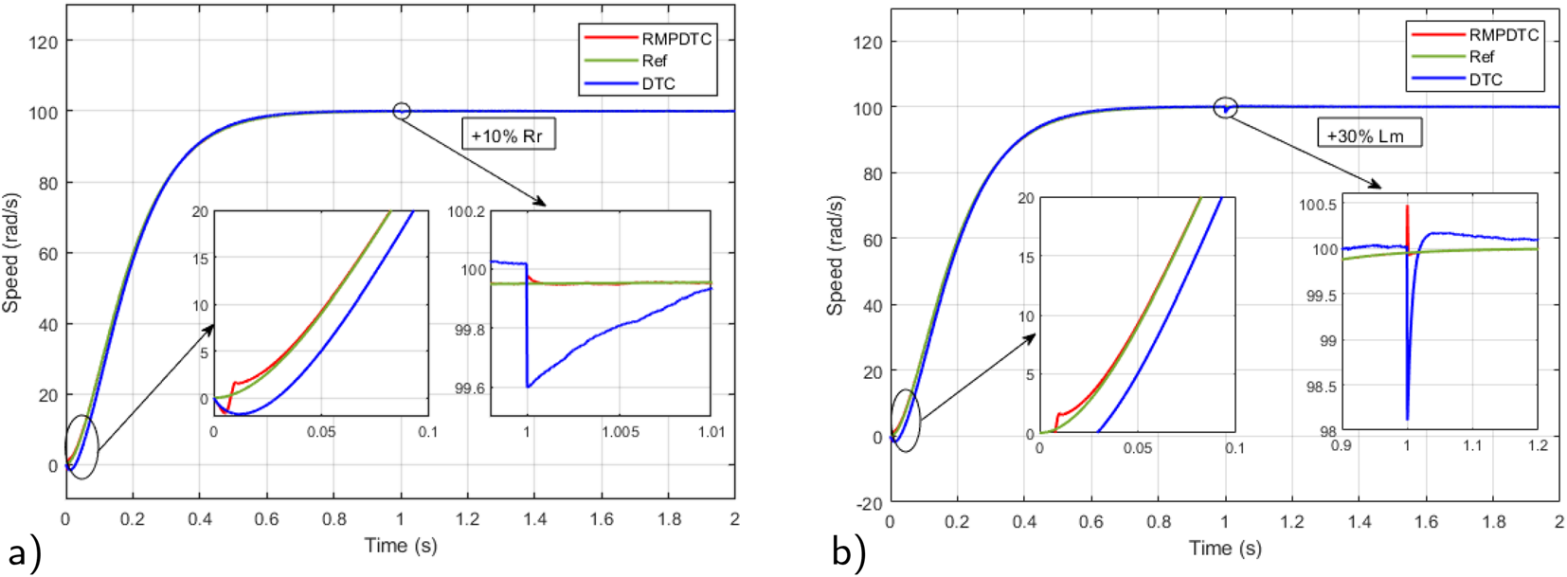
Simulation results for speed trajectory tracking under variation of: (**a**) $+10\%R_r$, (**b**) $+30\%L_m$.

In Fig. 14, the simulation results for speed trajectory tracking are presented for both the traditional DTC and the proposed RMPDTC. The RMPDTC effectively mitigates the effects of parametric variations, ensuring that the speed closely adheres to its reference point with minimal response time and no errors. This stands in contrast to the reference speed tracking observed in the classical DTC.

**Figure 15:**
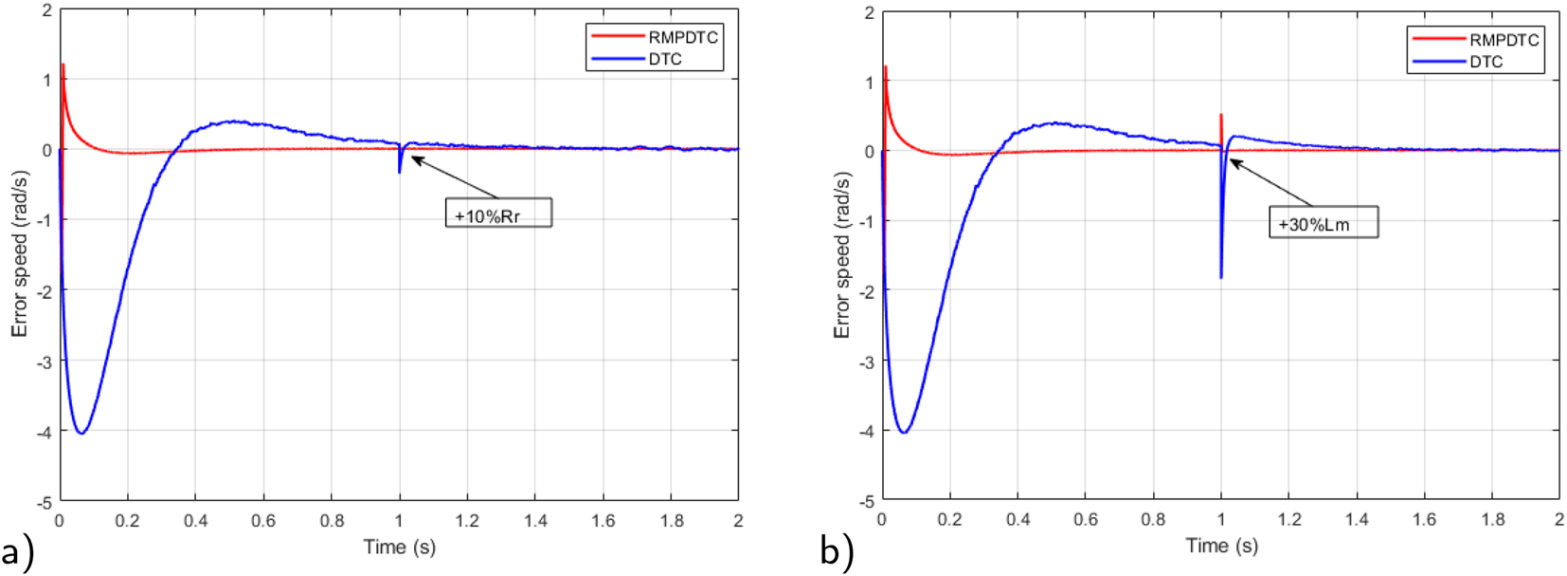
Simulation results for speed trajectory tracking error under variation of: (**a**) $+10\%R_r$, (**b**) $+30\%L_m$.

In Fig. 15, the simulation results depict the speed trajectory tracking error under variations in machine parameters in both the DTC and the proposed RMPDTC. The depicted graph of the proposed RMPDTC shows an error that swiftly converges to zero, ultimately reaching a stable state. This stands in contrast to the classical DTC, where the error gradually rises with any adjustments made to the machine settings.

**Figure 16:**
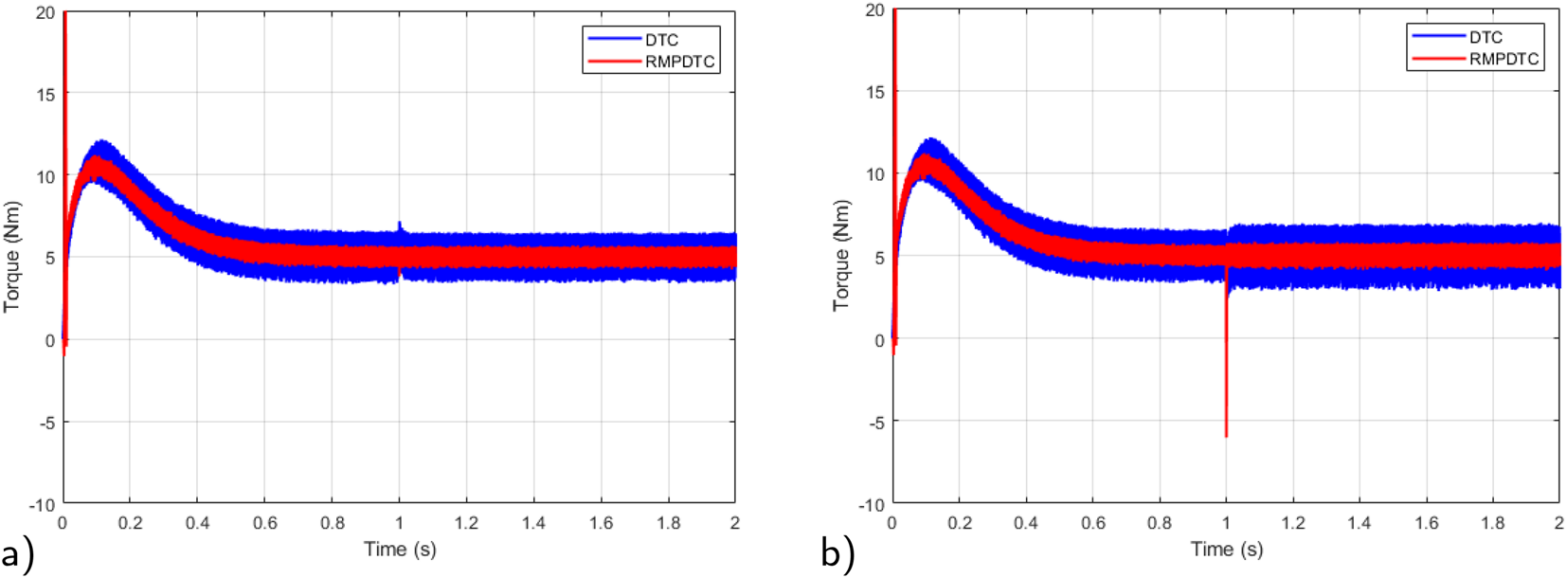
Simulation results of electromagnetic torque under variation of: (**a**) $+10\%R_r$, (**b**) $+30\%L_m$.

In Fig. 16, the simulation results depict the electromagnetic torque responses, and the results reveal a noticeably lower torque ripple in the RMPDTC compared to the classic DTC.

To assess the effectiveness of the controlled responses, two commonly used metrics, Integral Squared Error (ISE) and Integral Time-weighted Absolute Error (ITAE), have been employed. These metrics have been applied in association with the two control strategies, and the corresponding results are presented in Tables 4 and 5. The results from the experiments demonstrate a significant superiority in the outcomes achieved with the suggested approach in contrast to the traditional DTC. The adjustments implemented by the proposed strategy contribute to smoother transitions and reduced deviations from desired setpoints, ultimately resulting in an enhanced overall system performance. Additionally, the resilience of the proposed strategy becomes evident when facing challenges such as load torque disturbances and parameter variations. In contrast to the conventional DTC, the suggested strategy exhibits a heightened level of robustness.

**Table 4:** Comparative analysis using ISE metric.

	DTC	RMPDTC
Variation of $T_L$	4.235	0.014
Variation of $\alpha$	2.793	0.0071
Variation of $R_r$	2.203	0.023
Variation of $L_m$	2.2187	0.023

**Table 5:** Comparative analysis using ITAE metric.

	DTC	RMPDTC
Variation of $T_L$	10.085	0.287
Variation of $\alpha$	5.705	0.0474
Variation of $R_r$	0.202	0.005
Variation of $L_m$	0.241	0.004

**Figure 17:**
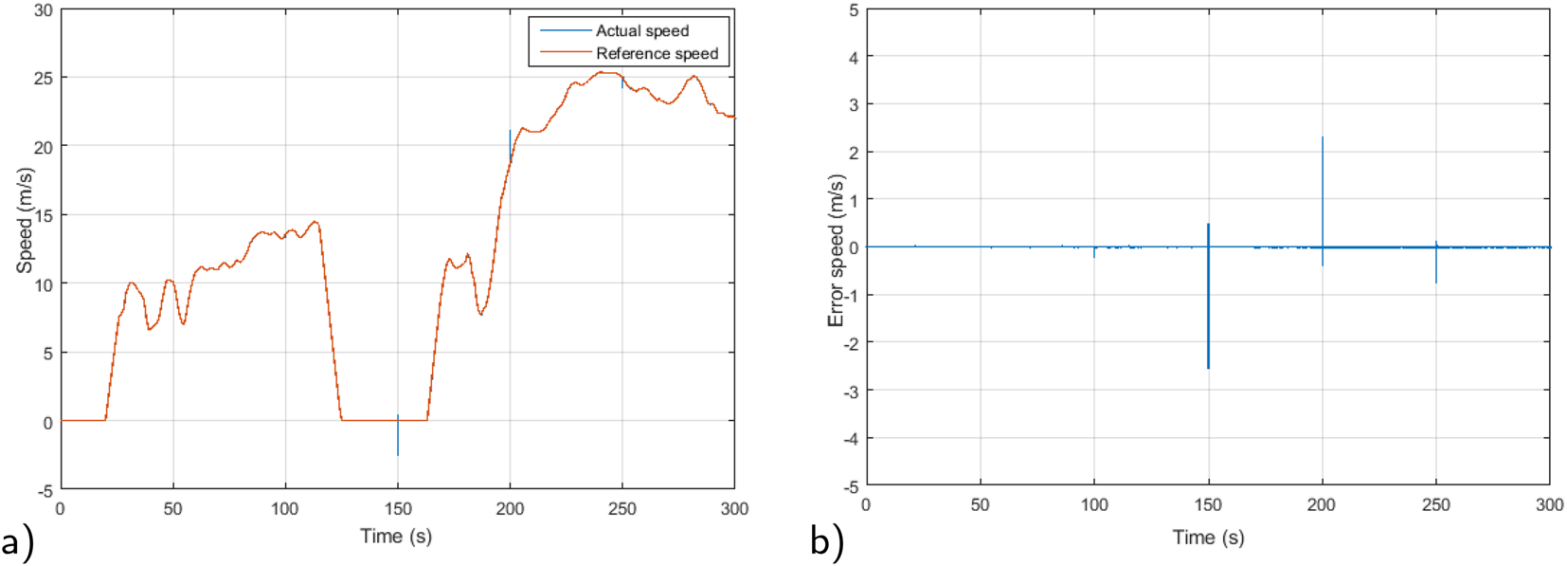
Performances of the EV traction chain under FTP-75 cycle. (**a**) Speed, (**b**) Error speed.

### RMPDTC strategy in electric vehicle application

The proposed RMPDTC system underwent simulation and evaluation under FTP-75 cycle conditions. The assessment included scenarios under load torque and parameters variations: stator resistance increased by $+10\%$, along with increments in the friction coefficient and flux by $+30\%$ at $t=150$ s, $t=200$ s, and $t=250$ s respectively. Figure 17a depicts the vehicle’s speed tracking. Despite the load torque disturbances and parameter fluctuations, the results reveal that the system tracks effectively and corresponds to the design criteria. The speed tracking error is presented in Fig. 17b. The results reveal that the error in the speed tracking is maintained at zero. Derived from these findings, it can be concluded that the control system adequately meets the requirements.

### Discussion and comparison

The technical literature has investigated multiple approaches to improve DTC performance under different speed and torque conditions, as summarized by the latest methods presented in Table 6. In^24^, a novel speed estimation method is introduced using an MRAS optimized by a genetic algorithm (GA). This method involves replacing the traditional MRAS’s PI controller with a GA-optimized adaptation mechanism. Nevertheless, while it shows disturbance rejection, its performance is still inferior to other controllers discussed in the literature. Additionally, the robustness against parametric disturbances was not addressed. In^26^, an ant colony optimization (ACO) algorithm was introduced to tune the PID controller gains of the DTC control. This approach employs a combined weighting cost function to achieve efficient and robust control. However, the reliance on hysteresis controllers leads to significant fluctuations in both flux and electromagnetic torque, these fluctuations also cause variable switching frequencies. In^33^, the authors introduced a space vector modulation approach integrated with Direct Torque Control (DTC-SVM). This method replaces hysteresis controllers with PI controllers. While striving to retain the advantages of DTC, this modification encounters notable hurdles, particularly stemming from the use of PI regulators. Challenges arise from frequent and significant variations in load torque, compounded by uncertainties in model parameters, which can lead to system instability. The RMPDTC method introduced in this study showcases remarkable capabilities in perturbation rejection, managing uncertainties in both electrical and mechanical parameters, and diminishing torque ripples. Given the prevalence of model parameter uncertainties in EV applications, any inaccuracies in system modeling or discrepancies in parameters can significantly affect control accuracy and even lead to system instability. Recognizing these obstacles, embracing the novel RMPDTC approach presents an enticing prospect for robust controller design, potentially surpassing conventional strategies.

**Table 6:** Comparison of the proposed approach with approaches utilized in prior studies.

Publication reference	Approaches	Response time (s)	Torque ripple (N m)	Robustness
El Ouanjli et al.^24^	GA-MRAS	0.124	0.7	Medium
Mahfoud et al.^26^	ACO-DTC	0.0256	1.91	High
Mahfoud et al.^33^	SVM-DTC	0.16	1.64	Low
Proposed	RMPDTC	0.08	0.5	High

## Experimental validation using the OPAL-RT platform

**Figure 18:**
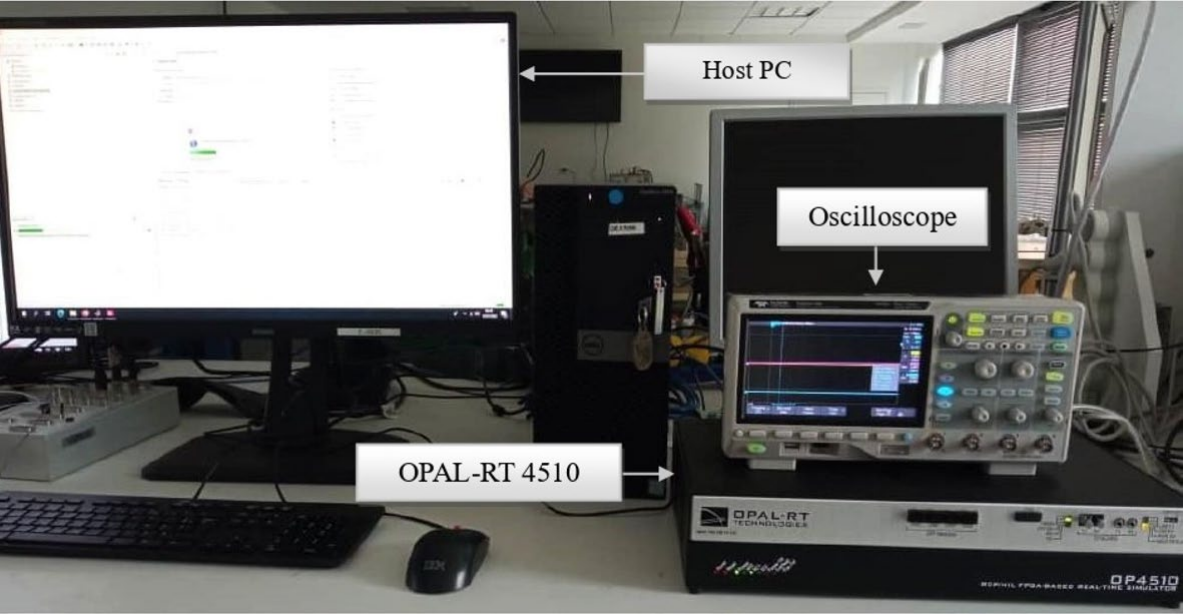
The real-time experimental setup.

In this part, we simulate the controlling system in real-time by utilizing the discrete real-time simulator known as the OPAL-RT platform. The configuration of the real-time simulation setup located at the French Naval Academy Research Institute is illustrated in Fig. 18. This setup comprises essential components, including a host PC, an OPAL-RT 4510 simulator, and an oscilloscope. The OPAL-RT platform is based on the OP4510, a comprehensive simulation system that can be executed on Kintex-7 FPGA systems. It operates on the Linux OS and is equipped with four active Intel Xeon E5 processor cores running at 3.2 GHz.

**Figure 19:**
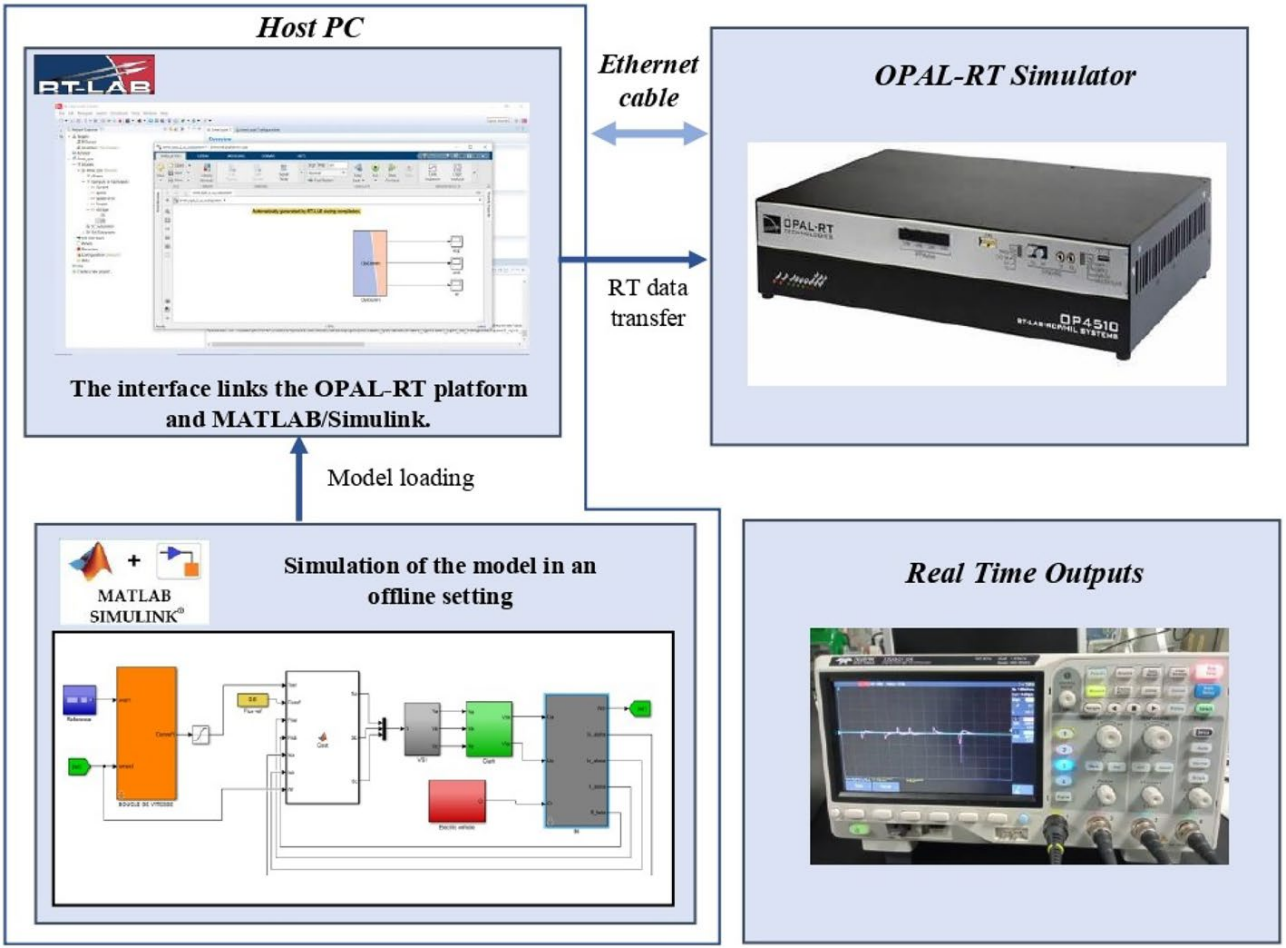
Process of real-time simulation platform.

The RT simulation process is illustrated in Fig. 19. Interaction between the OPAL-RT platform and MATLAB/Simulink occurs through the RT-LAB software. This software is seamlessly integrated with MATLAB/Simulink, enabling communication between Simulink models and the actual environment in real-time. Within the Simulink model, it is necessary to establish a subsystem that encompasses all power and control elements, alongside another subsystem that integrates display components. The RT-LAB software is employed to construct and compile the configured model into executable codes. These codes are then loaded and executed on multiple powerful processors within the OP4510 platform.

**Figure 20:**
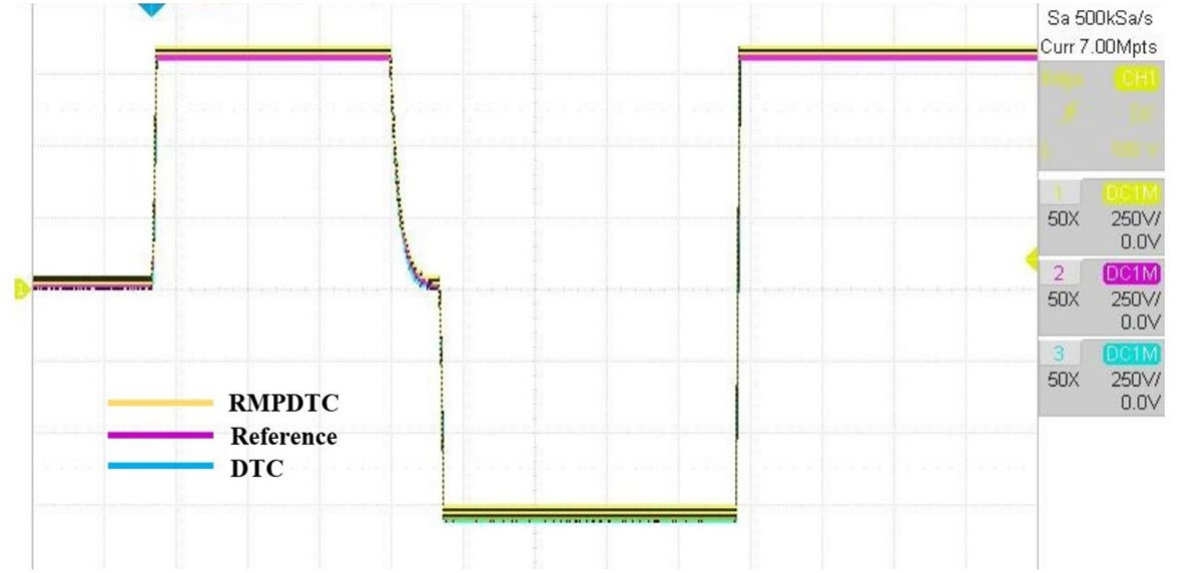
The experimental responses of actuals and reference speed under $T_L$ variation.

**Figure 21:**
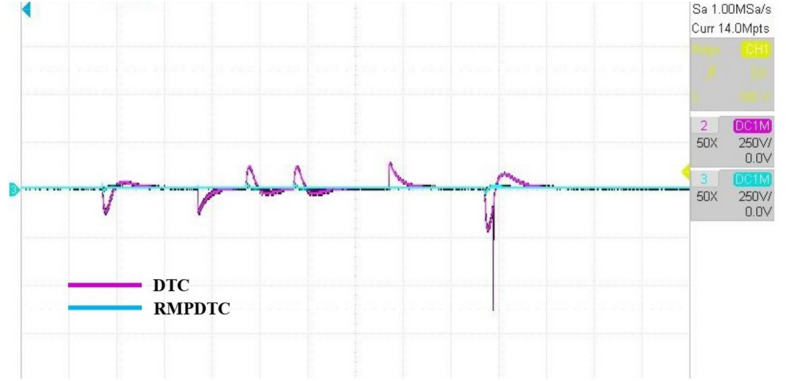
The experimental responses of the tracking speed error under $T_L$ variation.

**Figure 22:**
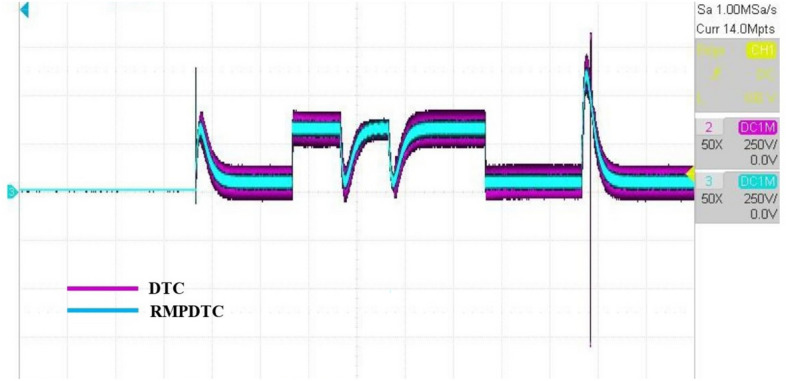
The experimental responses of electromagnetic torque under $T_L$ variation.

**Figure 23:**
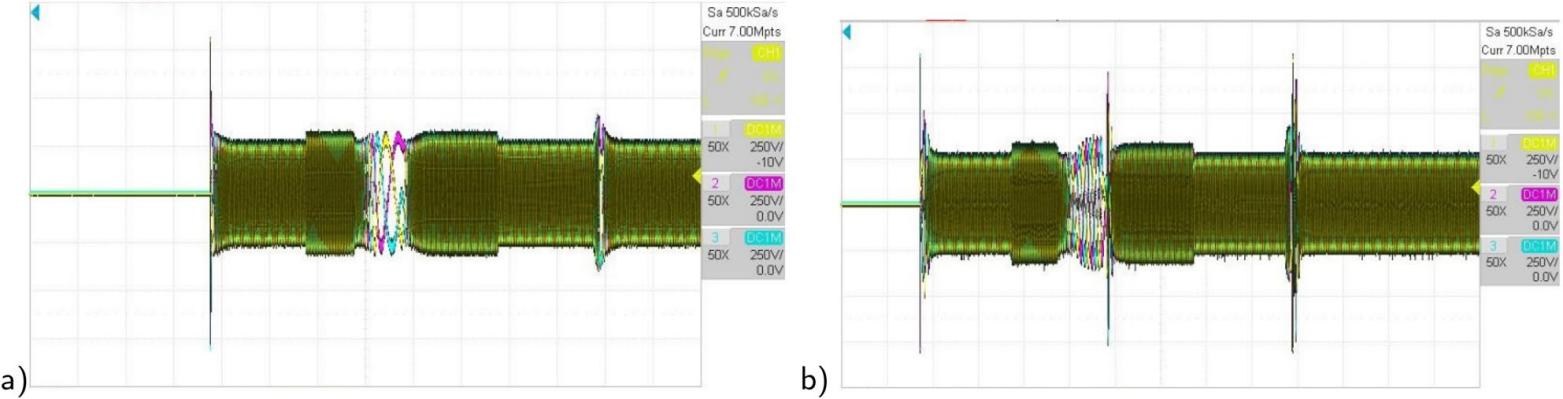
The experimental responses of three-phase stator currents under $T_L$ variation (**a**) Proposed RMPDTC (**b**) Classic DTC.

**Figure 24:**
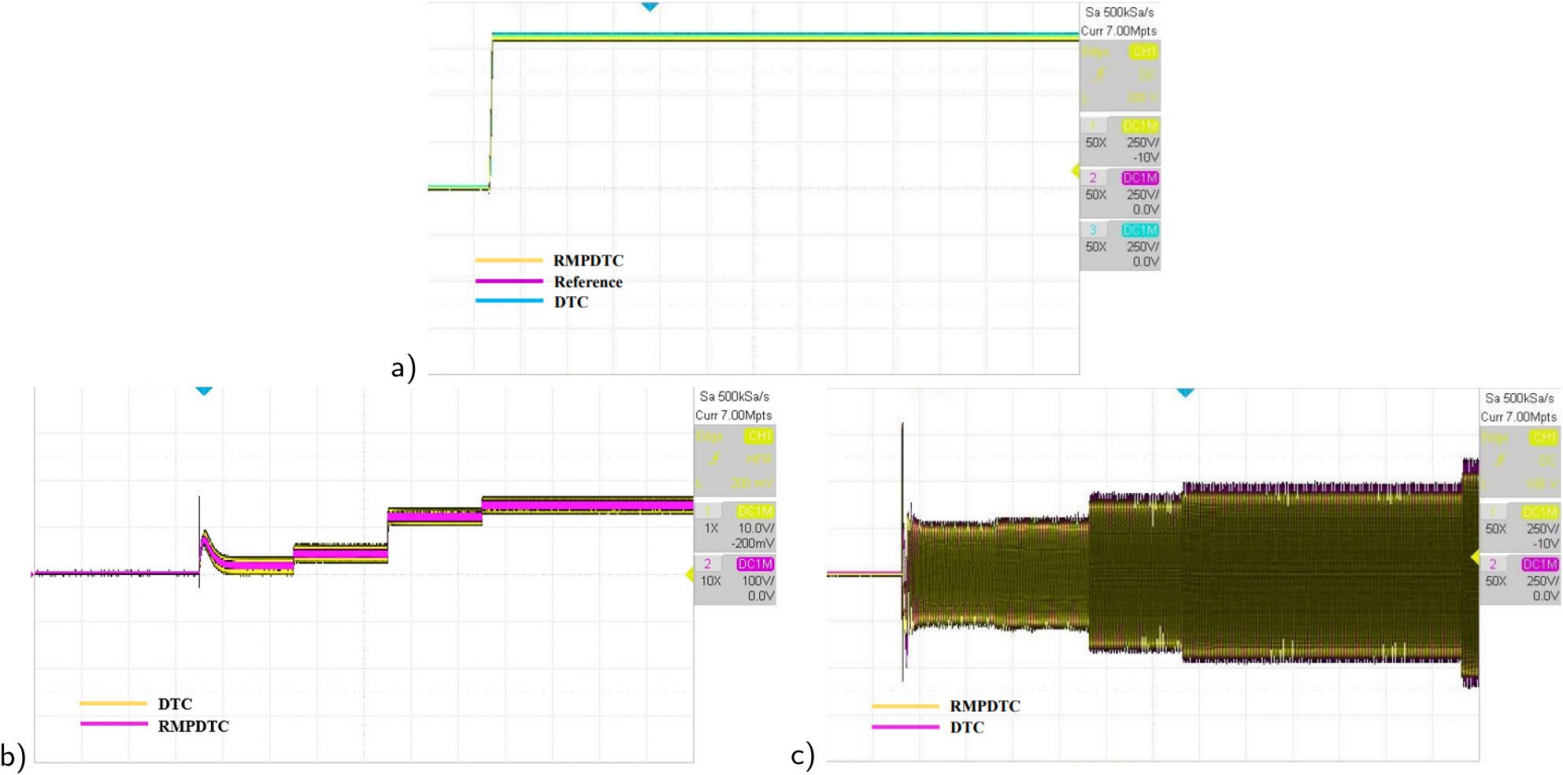
The experimental results under grade angle variations (**a**) speed, (**b**) torque, (**c**) stator phase current $(i_{sa})$.

**Figure 25:**
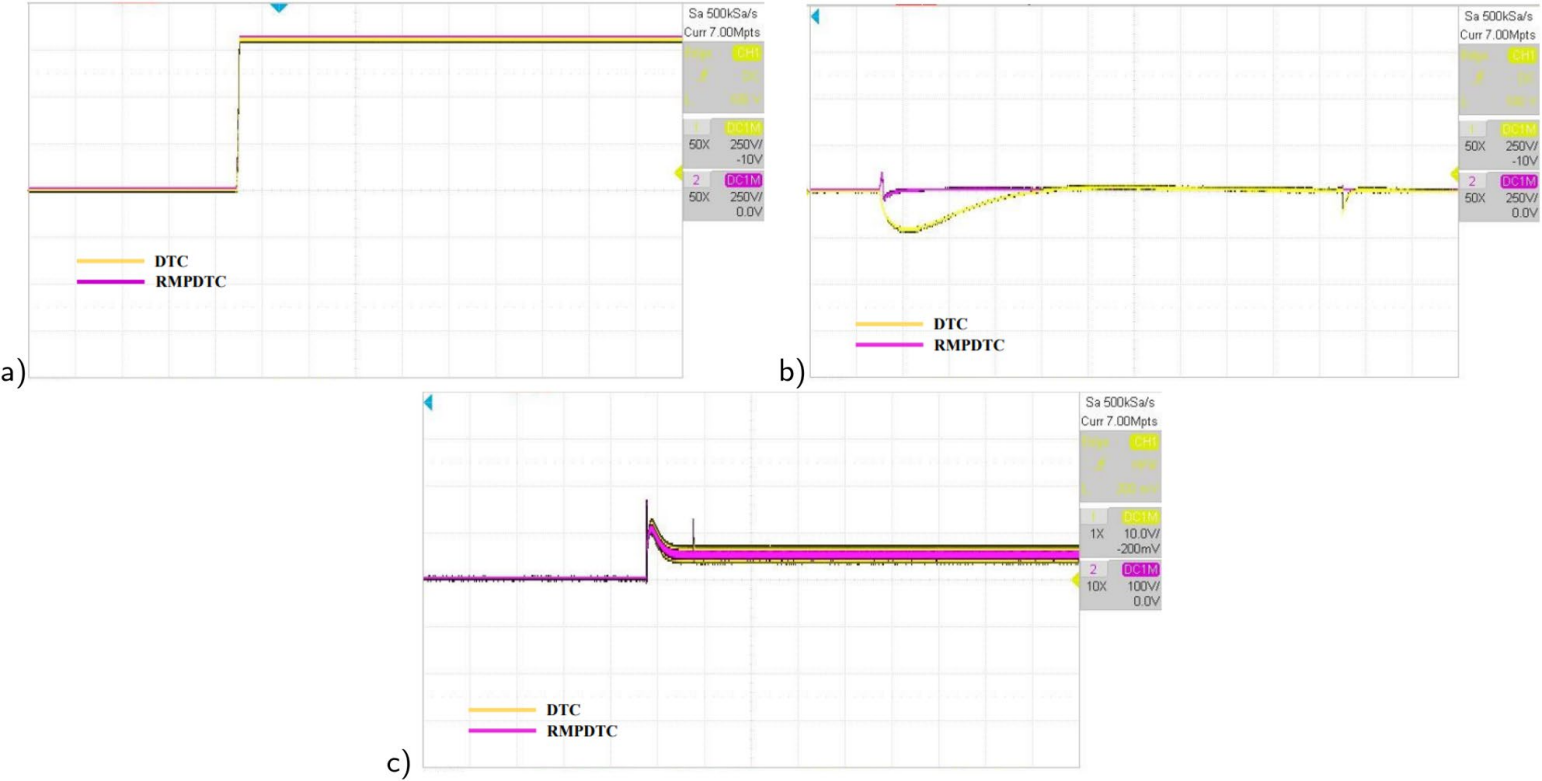
The experimental results under variation of $R_r$ (**a**) speed, (**b**) error speed, (**c**) torque.

The first assessment involves examining the controllers’ performance when subjected to load torque perturbations, and the experimental findings are depicted in Figs. 20, 21, 22 and 23. As depicted in Figs. 20 and 21, the proposed controller ensures precise tracking of the reference by accurately regulating the motor’s speed, exhibiting rapid dynamic response, and the error is effectively eliminated, converging to 0. Furthermore, the suggested RMPDTC demonstrates superior dynamics in minimizing overshoot values when altering the direction of the machine’s rotation. This is in contrast to the behavior observed in DTC, where the error tends to increase with each fluctuation in load torque and speed reversion. Figure 22 illustrates the electromagnetic torque generated by IM using both control systems. RMPDTC offers significant advantages, notably in the substantial reduction of electromagnetic torque ripple when compared to classical DTC. This positions the new controller as a preferred choice for minimizing the chattering phenomenon. Figure 23 depicts the experimental results of the stator current supplied to the induction motor. The magnitude of the stator current is closely associated with the electromagnetic torque, and it is evident that the proposed RMPDTC results in a noticeable decrease in peak current compared to the traditional DTC.

In the second evaluation, depicted in Fig. 24, it is evident that the suggested method effectively sustains the desired motor speed despite changes in the grade angle. This results in robust tracking performance in the presence of slope variations. Fig. 24b illustrates that the RMPDTC exhibits a significant decrease in torque ripples and achieves a swift torque response in comparison to the conventional DTC. Figure 24c demonstrates the stator phase current $(i_a)$ profiles for both DTC and RMPDTC. In contrast to the suggested approach, the conventional DTC exhibits a conspicuous high-magnitude sinusoidal waveform, indicating a significant degree of ripple.

To assess the effectiveness of the suggested approach amidst variations in motor parameters, Fig. 25 shows the experimental outcomes observed when comparing the conventional DTC with the newly proposed scheme. The rotor resistance ($R_r$) is selected as the parameter under examination. Real-time testing is conducted to evaluate sensitivity to parameter variations, specifically with a $+10 \%$ change from the initial value. In Fig. 25a, it is evident that even in the presence of fluctuations in the induction motor parameters, the system consistently reaches the predetermined reference value in its response. Notably, the speed precisely follows its reference trajectory. As shown in Fig. 25b, the error plot of the proposed RMPDTC exhibits rapid convergence to 0, in contrast to DTC where the error increases with variations in $R_r$. As evident from Fig. 25c, the utilization of RMPDTC results in a notable reduction in ripples in the electromagnetic torque.

The outcomes achieved through a real-time simulator, OPAL-RT, closely align with the simulation results, incorporating the same observations as highlighted earlier in the simulation findings. The experimental results provide evidence affirming the efficacy of the suggested control approach.

## Conclusion

This study focused on improving the performance of electric vehicles by incorporating a robust predictive controller for the regulation of speed and torque of induction motors. The proposed controller aims to enhance the performance of the conventional DTC approach. To achieve this, the traditional hysteresis comparators and switching tables were replaced with a predictive block based on an optimization algorithm. Additionally, a two-step forward prediction algorithm was employed to compensate for time delays. The speed regulation control system was improved by incorporating a novel robust finite horizon cost function, eliminating the need to measure and observe external disturbances. The proposed approach ensures effective tracking of speed trajectories, achieves an $84\%$ improvement in disturbance rejection compared to conventional DTC, enhances dynamic response by $70\%$, reduces torque ripple by $59\%$, and ensures overall robustness. Simulation results obtained through a discrete real-time simulator, validated on the OPAL-RT platform, affirmed the efficacy and resilience of the proposed RMPDTC.

## Data Availability

The datasets used and/or analysed during the current study available from the corresponding author on reasonable request.

## List of symbols

DTC: Direct torque control
FOC: Field oriented control
IM: Induction machine
MPC: Model predictive control
RMPDTC: Robust model predictive direct torque control
ITAE: Integral time-weighted absolute error
ISE: Integral squared error
$i_{s\alpha }$, $i_{s\beta }$: Stator currents in the reference frame ($\alpha , \beta$)
$\psi _{s\alpha }$, $\psi _{s\beta }$: Flux in the reference frame ($\alpha , \beta$)
$v_{s\alpha }$, $v_{s\beta }$: Stator voltage in the reference frame ($\alpha , \beta$)
$L_s,L_r$: Stator and rotor inductance
$R_s$, $R_r$: Stator and rotor resistance
$$T_{em}$$: Electromagnetic torque
$$T_L$$: Load torque
*p*: Number of pole pairs
*f*: Coefficient of the viscous friction
*J*: Inertia moment
$\rho$: Relative degree of output
$\omega _r$: Electrical rotor speed
*M*(*x*): Decoupling matrix
$y_{ref} (t)$: Trajectory of reference
*y*(*t*): System output
$\Pi (t)$: The error integral
$\Pi (t+\tau )$: The prediction of the error integral
*x*(*t*): State vector
*u*(*t*): Control vector
$S_a, S_b, S_c$: The switching state
